# Role of STAT3‐FOXO3 Signaling in the Modulation of Neuroplasticity by PD‐L1‐HGF‐Decorated Mesenchymal Stem Cell‐Derived Exosomes in a Murine Stroke Model

**DOI:** 10.1002/advs.202404882

**Published:** 2024-07-25

**Authors:** Syuan‐Ling Lin, Yi‐Wen Chang, Wei Lee, Chih‐Sheng Chiang, Shih‐Ping Liu, Hsu‐Tung Lee, Long‐Bin Jeng, Woei‐Cherng Shyu

**Affiliations:** ^1^ Translational Medicine Research Center and Department of Neurology China Medical University Hospital Taichung 404 Taiwan; ^2^ Cell Therapy Center China Medical University Hospital Taichung 404 Taiwan; ^3^ Department of Medical Research National Taiwan University Hospital Taipei 100 Taiwan; ^4^ Graduate Institute of Biomedical Sciences and New Drug Development Center China Medical University Taichung 404 Taiwan; ^5^ Graduate Institute of Medical Sciences National Defense Medical Center Taipei 114 Taiwan; ^6^ Department of Post‐Baccalaureate Medicine, College of Medicine National Chung Hsing University Taichung 402 Taiwan; ^7^ Division of neurosurgical Oncology Neurological Institute Taichung Veterans General Hospital Taichung 407 Taiwan; ^8^ Organ Transplantation Center China Medical University Hospital Taichung 404 Taiwan; ^9^ Department of Occupational Therapy Asia University Taichung 413 Taiwan

**Keywords:** exosomes, HGF, PD‐L1, STAT3‐FOXO3 pathway, stroke

## Abstract

The limited therapeutic strategies available for stroke leave many patients disabled for life. This study assessed the potential of programmed death‐ligand 1 (PD‐L1) and hepatocyte growth factor (HGF)‐engineered mesenchymal stem cell‐derived exosomes (EXO‐PD‐L1‐HGF) in enhancing neurological recovery post‐stroke. EXO‐PD‐L1‐HGF, which efficiently endocytosed into target cells, significantly diminishes the H_2_O_2_‐induced neurotoxicity and increased the antiapoptotic proteins in vitro. EXO‐PD‐L1‐HGF attenuates inflammation by inhibiting T‐cell proliferation and increasing the number of CD8^+^CD122^+^IL‐10^+^ regulatory T cells. Intravenous injection of EXO‐PD‐L1‐HGF could target stromal cell‐derived factor‐1α (SDF‐1α^+^) cells over the peri‐infarcted area of the ischemic brain through CXCR4 upregulation and accumulation in neuroglial cells post‐stroke. EXO‐PD‐L1‐HGF facilitates endogenous nestin^+^ neural progenitor cell (NPC)‐induced neurogenesis via STAT3‐FOXO3 signaling cascade, which plays a pivotal role in cell survival and neuroprotection, thereby mitigating infarct size and enhancing neurological recovery in a murine stroke model. Moreover, increasing populations of the immune‐regulatory CD19^+^IL‐10^+^ and CD8^+^CD122^+^IL‐10^+^ cells, together with reducing populations of proinflammatory cells, created an anti‐inflammatory microenvironment in the ischemic brain. Thus, innovative approaches employing EXO‐PD‐L1‐HGF intervention, which targets SDF‐1α^+^ expression, modulates the immune system, and enhances the activation of resident nestin^+^ NPCs, might significantly alter the brain microenvironment and create a niche conducive to inducing neuroplastic regeneration post‐stroke.

## Graphical abstract

The study shows the therapeutic potential of programmed death‐ligand 1 (PD‐L1) and hepatocyte growth factor (HGF)‐engineered human telomerase reverse transcriptase‐immortalized adipose tissue‐derived mesenchymal stem cell‐derived exosomes (EXO‐PD‐L1‐HGF) in promoting neuroprotection, anti‐inflammation, and neurogenesis following ischemic stroke. By exploring the molecular mechanism of STAT3‐FOXO3 signaling axis, the novel cell‐free therapeutic product, EXO‐PD‐L1‐HGF‐activated endogenous nestin^+^ NPCs, might augment neurological functional recovery post‐stroke.

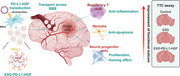

## 1 Introduction

Stroke is the second leading cause of death worldwide.^[^ ^1^ ^]^ Ischemic stroke is a medical emergency that occurs when blood vessels are occluded. Of note, the sequelae of stroke, including paralysis, depression, speech disturbance, and visual disturbance, remain in ≈75% of stroke survivors.^[^ ^2^ ^]^ Although thrombolytic therapy with tissue plasminogen activator and endovascular thrombectomy for acute ischemic stroke are available with a short therapeutic window,^[^ ^3^ ^]^ more than 1000 new drugs clinical trials have failed to develop safe and effective therapies for stroke. Consequently, there is a need to develop effective therapeutic strategies to improve brain recovery in patients with stroke and other disabilities.

Mesenchymal stem cells (MSCs) are one of the most commonly used cell types for cell‐based therapies in regenerative medicine and immune therapy,^[^ ^4^ ^]^ to improve neurological function in cerebrovascular disease.^[^ ^5^ ^]^ Owing to their immunomodulatory properties, MSCs suppress the activation of various immune effector cells and promote immune‐regulatory functions.^[^ ^6^ ^]^ MSCs are multipotent adult stem cells capable of self‐renewal and multilineage differentiation. As paracrine signaling is the mechanism of action underlying the therapeutic efficacy of MSCs,^[^ ^7^ ^]^ MSC‐derived exosomes are generally easier to manufacture and safer (without unwanted engraftment) than cell‐based therapies. Exosomes are a category of nanoparticles with diameters in the range of 30–200 nm that are used to enclose many bioactive molecules such as lipids, proteins, messenger RNAs (mRNAs), long noncoding RNAs (lncRNAs), and microRNAs (miRNAs).^[^ ^8^ ^]^ These biomolecules are distinct payloads determined by the parent cell, which is the cell that generated the exosomes. Acting as messengers, exosomes carry these biomolecules to recipient cells, where they are selectively absorbed to influence various cellular activities, including gene expression, cell communication, immune responses, and tissue repair. Additionally, due to their natural origin, nanoscale size, low immunogenicity, and capacity to traverse various biological barriers, exosomes are regarded as promising drug carriers for targeted therapies. Therefore, MSC‐derived exosomes play an important role in promoting neural plasticity and neurological functional recovery in various central nervous system disease models.^[^ ^9^ ^]^

It is well known that programmed death‐1 (PD‐1), which is expressed on the cell surface of activated T and B cells, interacts with its ligand, programmed death‐ligand 1 (PD‐L1), to provide an inhibitory signal for the regulation of cellular activation and proliferation.^[^ ^10^ ^]^ The induction of signaling through the PD‐1 and PD‐L1 pathways causes T‐cell exhaustion, which in turn suppresses the inflammatory cascade in the central nervous system in a murine stroke model.^[^ ^11^ ^]^ Furthermore, hepatocyte growth factor (HGF) treatment enhances proliferation of neural precursor cells (NPCs) and segmented neurovascular remodeling to induce long‐term neuroprotection in animals with stroke.^[^ ^12^ ^]^ In view of the above evidence, the combination of HGF and PD‐L1 could serve as an innovative genetic engineering candidate in developing therapeutic interventions for neurogenesis and inflammation.

Augmented proliferation of endogenous nestin^+^ NPCs in subventricular zone (SVZ) neurogenic niches^[^ ^13^ ^]^ substantially contributes to neuroplasticity. During stroke induction, a significant population of nestin^+^ NPCs within the SVZ are diverted from their initial migration route toward the peri‐infarct region, signifying a possible role for them in poststroke recovery.^[^ ^14^ ^]^ FOXO3 modulates the homeostasis of NPCs, to preserve cell quiescence and prevent premature differentiation, by inducing a program of self‐renewal genes.^[^ ^15^ ^]^ Upregulation of FOXO3 induction by hypoxia‐ischemia^[^ ^16^ ^]^ is linked to the hypothesis that activating FOXO3 expression using a specific therapeutic strategy can promote the proliferation of NPCs for recovery poststroke.

Since MSCs possess the hereditary expression of PD‐L1 and HGF, genetically engineering these cells to overexpress the two molecules might be a good strategy for rescuing the neuroglial tissue injured by stroke, to enhance treatment efficacy. In this study, we investigated the therapeutic potential of EXO‐PD‐L1‐HGF in promoting neuroprotection, anti‐inflammation, and neurogenesis following ischemic stroke. By exploring the molecular mechanism of STAT3‐FOXO3 signaling axis, the novel cell‐free therapeutic product, EXO‐PD‐L1‐HGF‐activated endogenous nestin^+^ NPCs, might augment neurological functional recovery after ischemic stroke.

## 2 Results

### 2.1 Isolation and Characterization of PD‐L1‐HGF‐Modified Human Telomerase Reverse Transcriptase‐Immortalized Adipose Tissue‐Derived Mesenchymal Stem Cell (hTERT‐ADSC)‐Derived Exosomes

**Figure 1:**
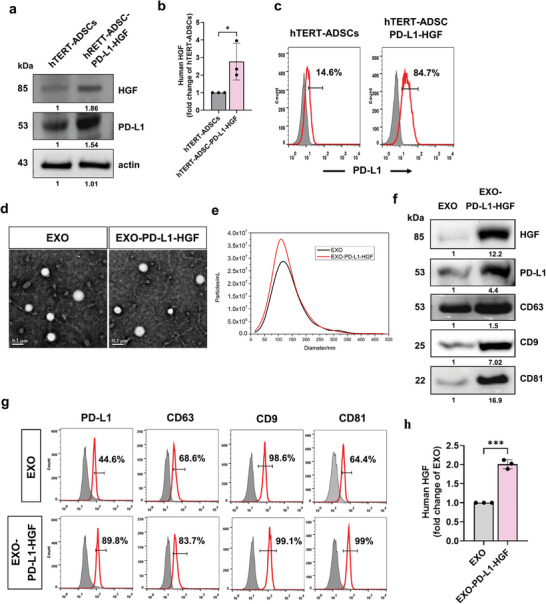
Characterization of PD‐L1‐HGF‐modified hTERT‐ADSC‐derived exosomes. a) Western blot, b) ELISA, and c) flow cytometry of hTERT‐ADSC‐PD‐L1‐HGF cells overexpressing HGF and/or PD‐L1. d) Assessment of exosome morphology by means of transmission electron microscopy. Scale bar: 100 nm. e) Particle size distribution of exosomes, as measured using nanoparticle tracking analysis. f) Western blot and g) flow cytometry of specific exosomal surface markers CD63, CD9, and CD81, as well as the transgenic markers HGF and PD‐L1, in the EXO‐ and EXO‐PD‐L1‐HGF‐treated groups. h) ELISA for the HGF concentration in the EXO‐ and EXO‐PD‐L1‐HGF‐treated groups. Data are presented as mean ± standard deviation (SD) (*p < 0.05; **p < 0.01). ELISA, enzyme‐linked immunosorbent assay; PD‐L1, programmed death‐ligand 1; CD, cluster of differentiation; HGF, hepatocyte growth factor; hTERT‐ADSC, human telomerase reverse transcriptase‐immortalized adipose tissue‐derived mesenchymal stem cell.

To investigate the efficacy of PD‐L1‐HGF‐modified exosomes derived from hTERT‐ADSCs (EXO‐PD‐L1‐HGF) in the treatment of ischemic stroke, we used an electroporation system to transduce the PiggyBac (PB)‐CMV‐PD‐L1‐HGF plasmid into hTERT‐ADSCs and isolate EXO‐PD‐L1‐HGF. To verify that genetic manipulation does not disturb the stemness of MSCs, we first assessed the differentiation potential of hTERT‐ADSC‐PD‐L1‐HGF into osteoblasts, chondrocytes, and adipocytes and compared it with that of the parent hTERT‐ADSCs and primary ADSCs (Figure S1a, Supporting Information). Western blot showed that PD‐L1 and HGF were significantly overexpressed in hTERT‐ADSC‐PD‐L1‐HGF than in hTERT‐ADSCs, thereby validating the successful electroporation of the plasmid (**Figure** **1**a). Enzyme‐linked immunosorbent assay (ELISA) showed that the hTERT‐ADSC‐PD‐L1‐HGF cells displayed a ≈1.5‐fold higher HGF concentration than hTERT‐ADSCs (Figure 1b). Moreover, flow cytometry showed that the surface expression of PD‐L1 was upregulated in hTERT‐ADSC‐PD‐L1‐HGF cells (by ≈85%) (Figure 1c). We optimized the production and isolation of exosomes from the supernatants of hTERT‐ADSC‐PD‐L1‐HGF and hTERT‐ADSC cells, to increase their yields of EXO‐PD‐L1‐HGF and EXO, respectively (Figure S1b, Supporting Information). Direct examination of the morphology of the isolated exosomes by means of transmission electron microscopy revealed a homogenous round membrane vesicle (Figure 1d). Nanoparticle tracking analysis revealed that the exosomes displayed a particle size distribution of 100–200 nm (Figure 1e). The increased protein expression of specific exosomal markers (such as CD9, CD63, and CD81), as well as transgenic markers (such as PD‐L1 and HGF), as assessed using western blot, further confirmed the identity of the exosomes and the direct inclusion of the PD‐L1/HGF proteins into EXO‐PD‐L1‐HGF, as compared with that into EXO (Figure 1f). Moreover, flow cytometry confirmed that EXO‐PD‐L1‐HGF caused a significant increase in PD‐L1, CD9, CD63, and CD81 levels than EXO (Figure 1g). Consistently, ELISA revealed that EXO‐PD‐L1‐HGF resulted in higher expression of HGF than EXO (Figure 1h).

### 2.2 EXO‐PD‐L1‐HGF Significantly Attenuated the H_2_O_2_‐Induced Neuronal Apoptosis In Vitro

**Figure 2:**
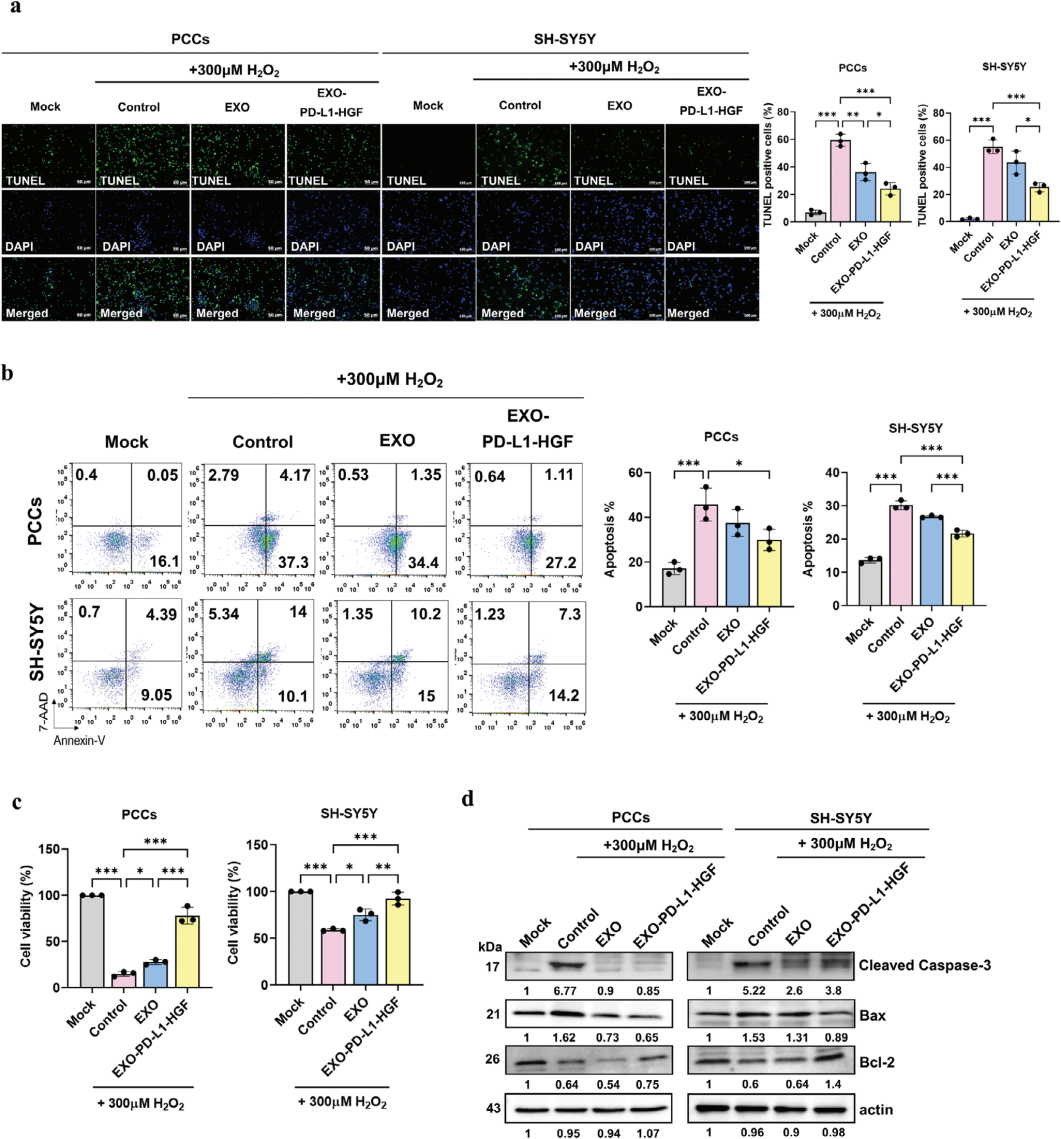
EXO‐PD‐L1‐HGF significantly attenuated the H_2_O_2_‐induced neuronal apoptosis in vitro. a) TUNEL staining was used to detect apoptosis in PCCs and SH‐SY5Y cells. Cell nuclei were counterstained with DAPI (blue). Quantitative estimation of the proportion of apoptotic cells in each of the following experimental groups: groups pretreated with mock, control, EXO, and EXO‐PD‐L1‐HGF, followed by 300 µm H_2_O_2_, as indicated. b) Annexin V‐FITC/7‐AAD double staining was used to detect cell apoptosis induced by H_2_O_2_ in the PCCs and SH‐SY5Y cells pretreated with mock, control, EXO, and EXO‐PD‐L1‐HGF. c) CCK‐8 assay was used to assess the cell viability of PCCs and SH‐SY5Y cells pretreated with mock, control, EXO, and EXO‐PD‐L1‐HGF overnight and then exposed to 300 µm H_2_O_2_. d) Western blot of proteins (e.g., Bax, caspase‐3, and Bcl‐2) involved in the H_2_O_2_‐induced apoptosis of PCCs and SH‐SY5Y cells. Data are presented as mean ± SD (*p < 0.05; **p < 0.01). TUNEL, terminal deoxynucleotidyl transferase dUTP nick‐end labeling; PD‐L1, programmed death‐ligand 1; HGF, hepatocyte growth factor; DAPI, 4′,6‐diamidino‐2‐phenylindole; CCK‐8, cell counting kit‐8; PCCs, primary cortical cells; H_2_O_2_, hydrogen peroxide.

We examined the cellular uptake of fluorescently labeled EXO‐PD‐L1‐HGF and EXO in vitro using immunofluorescence analysis with DiO, which gives off green fluorescence. After 1 h of incubation with PCCs, tiny green fluorescent spots of DiO‐EXO‐PD‐L1‐HGF and DiO‐EXO were endocytosed into the cytoplasm of MAP2‐expressing PCCs (Figure S2a, Supporting Information). To investigate whether EXO‐PD‐L1‐HGF attenuated H_2_O_2_‐induced cell death, we determined the optimal concentration and duration of H_2_O_2_ treatment by exposing PCCs and SH‐SY5Y cells to varying concentrations of H_2_O_2_ (0, 100, 200, 300, 400, and 500 µM) for 3 h. Cell viability was estimated using the cell counting kit‐8 (CCK‐8) assay and expressed as a percentage of the untreated control. H_2_O_2_ treatment for three hours concentration‐dependently induced significant cell death in both PCCs and SH‐SY5Y cells (Figure S2b, Supporting Information). Based on these results, we determined 300 µm as the optimal treatment dose for H_2_O_2_ in the subsequent experiments. Pretreatment with EXO‐PD‐L1‐HGF overnight significantly reduced the H_2_O_2_‐induced apoptotic ratio (TUNEL^+^ cells showed green fluorescence), as compared to that upon pretreatment with EXO and that in the control, in both PCCs and SH‐SY5Y cells, as determined using terminal deoxynucleotidyl transferase dUTP nick‐end labeling (TUNEL) assay (**Figure** **2**a). Consistently, pretreatment with EXO‐PD‐L1‐HGF significantly mitigated apoptosis after exposure to H_2_O_2_, as compared with that upon pretreatment with EXO and that in the untreated control, as shown by means of Annexin V‐ fluorescein isothiocyanate (FITC)/7‐aminoactinomycin D (7‐AAD) double staining (Figure 2b). Similarly, CCK‐8 assay determined a greater reduction in H_2_O_2_‐induced cell death after pretreatment with EXO‐PD‐L1‐HG than that after pretreatment with EXO and that in the control (≈80% of cell viability in PCCs and ≈85% in SH‐SY5Y cells) (Figure 2c). Furthermore, the expression levels of the proapoptotic proteins Bax and cleaved caspase‐3 were significantly decreased, while those of the antiapoptotic protein Bcl‐2 were elevated, in PCCs and SH‐SY5Y cells pretreated with EXO‐PD‐L1‐HGF for 24 h before H_2_O_2_ administration, as compared with those upon pretreatment with EXO and those in the control (Figures 2d and S3, Supporting Information). Taken together, these findings suggested that EXO‐PD‐L1‐HGF exerted neuroprotective effects against H_2_O_2_‐induced cytotoxicity.

### 2.3 EXO‐PD‐L1‐HGF Suppressed the Activation of Cytotoxic T Cells and Promoted That of Regulatory T (Treg) Cells

**Figure 3:**
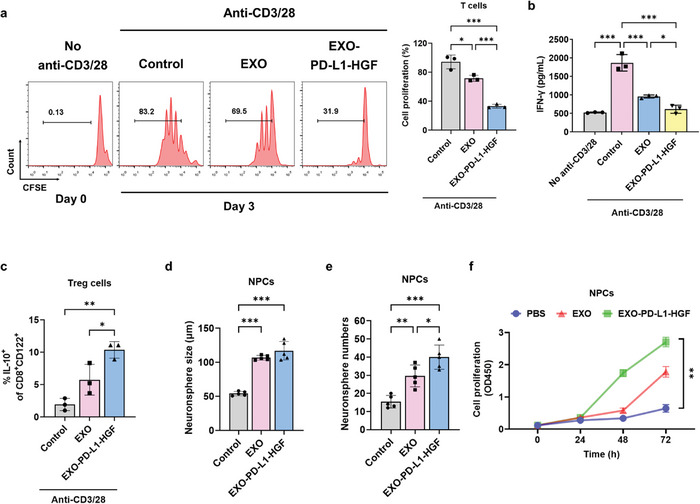
EXO‐PD‐L1‐HGF suppressed the activation of cytotoxic T cells, promoted the activation of Tregs, and enhanced the proliferation of NPCs. a) Purified CD3‐expressing T cells were cultured with an anti‐CD3/28 activator, stained with CFSE dye, and then treated with control, EXO, and EXO‐PD‐L1‐HGF for 3 d, following which the proliferation of the cells was assessed by means of flow cytometry. b) ELISA was used to detect the expression of the proinflammatory cytokine IFN‐γ in the control, EXO, and EXO‐PD‐L1‐HGF groups. c) Purified CD3‐expressing T cells were cultured with an anti‐CD3/28 activator and treated with control, EXO, and EXO‐PD‐L1‐HGF for 3 d. Flow cytometry showed that EXO‐PD‐L1‐HGF promoted the production of IL‐10 by CD8^+^CD122^+^ Treg cells. d,e) NPCs were seeded in an ultralow well plate and treated with control, EXO, and EXO‐PD‐L1‐HGF for 7 d. The size and number of NPCs were increased in the EXO‐PD‐L1‐HGF treatment group, as compared to that in the other groups. f) The EXO‐PD‐L1‐HGF group promoted the proliferation of NPCs, as shown using the CCK‐8 assay. NPCs, neural progenitor cells; CCK‐8, cell counting kit‐8; PD‐L1, programmed death‐ligand 1; Tregs, regulatory T cells; CFSE, carboxyfluorescein succinimidyl ester; CD, cluster of differentiation.

To investigate whether EXO‐PD‐L1‐HGF mitigates the expansion of T cells with a CD3/CD28 T‐cell activator in vitro, the fluorescence intensity of carboxyfluorescein succinimidyl ester (CFSE)‐labeled T cells was measured during T‐cell proliferation. Upon treatment of CFSE‐labeled proliferating T cells with control, EXO, or EXO‐PD‐L1‐HGF for 3 d, the percentage of T‐cell proliferation was significantly lower in the EXO‐PD‐L1‐HGF‐treated group than in the other groups (**Figure** **3**a). To assess whether EXO‐PD‐L1‐HGF affects the activation of T cells, we measured the interferon gamma (IFN‐γ) levels using ELISA, which indicated significantly reduced levels in the EXO‐PD‐L1‐HGF‐treated group than in the other groups (Figure 3b). Moreover, CD8^+^CD122^+^IL‐10^+^ Tregs were upregulated in the EXO‐PD‐L1‐HGF treatment group, as determined using flow cytometry (Figure 3c). Taken together, these results validated that intervention with EXO‐PD‐L1‐HGF could exert an immunosuppressive effect.

### 2.4 EXO‐PD‐L1‐HGF Promoted the Proliferation of Primary NPCs In Vitro

To investigate the effect of EXO‐PD‐L1‐HGF on the proliferation of NPCs, we isolated NPCs from cortical regions of embryos at embryonic day 17, as previously described,^[^ ^17^ ^]^ and analyzed the expression of Nestin and Sox2 in them by means of immunofluorescence analysis, to confirm their identity as neurospheres (Figure S4, Supporting Information). The EXO‐PD‐L1‐HGF treatment group exhibited significantly increased neurosphere‐forming ability than the EXO treatment group (Figure 3d,e). Next, examination of the proliferation of NPCs upon control, EXO, and EXO‐PD‐L1‐HGF treatment by means of CCK‐8 assay revealed higher proliferation in the EXO‐PD‐L1‐HGF‐treated group than in the EXO‐treated and control groups (Figure 3f). These results demonstrated that EXO‐PD‐L1‐HGF significantly promoted the proliferative ability of NPCs.

### 2.5 EXO‐PD‐L1‐HGF Positively Regulated the STAT3/FOXO3 Signaling Pathway Involved in NPCs‐Induced Neuroregeneration

**Figure 4:**
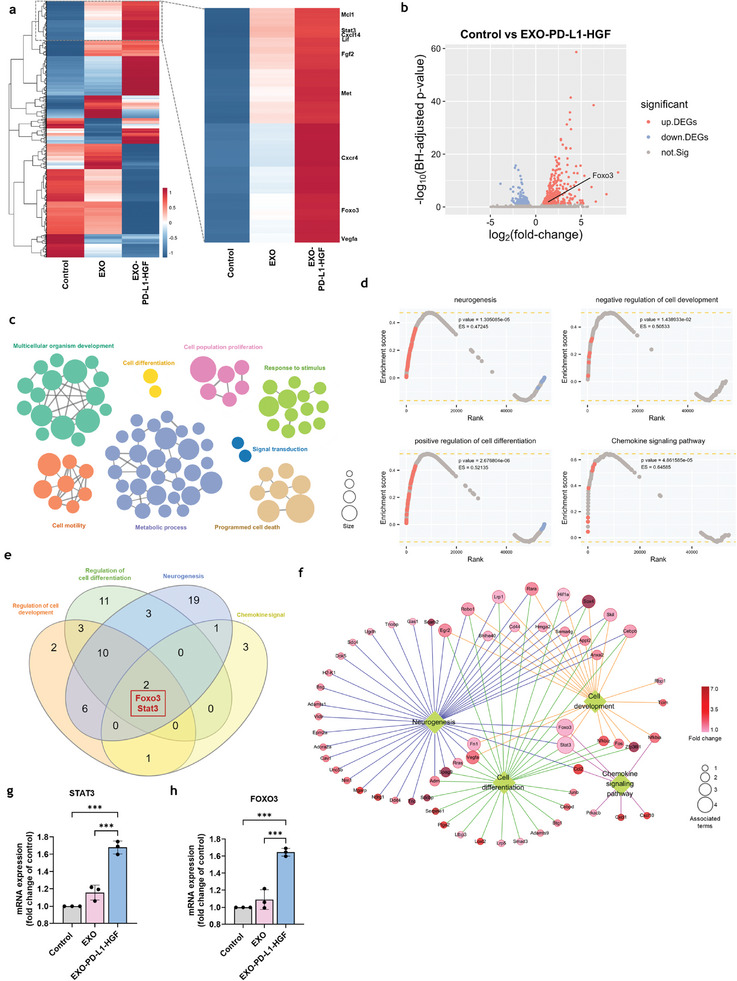
Identification of differentially expressed genes in NPCs treated with EXO and EXO‐PD‐L1‐HGF. a) The clustering heatmaps showed that several neurogenesis‐related genes, including MET, VEGF, bFGF, CXCR4, STAT3, and FOXO3, displayed upregulation in the EXO‐PD‐L1‐HGF treatment group. b) Comparison of the expression profiles of cells revealed that 311 genes were upregulated upon EXO‐PD‐L1‐HGF treatment, as compared with that upon the EXO‐PD‐L1‐HGF and control treatments, using fold‐change >1.5 and an adjusted *p*‐value < 0.05 as criteria. c) GO annotation revealed that these differentially expressed genes were involved in functions related to cell development, cell differentiation, response to stimulus, and cell motility. d) GSEA using both GO and KEGG pathway databases indicated that genes mediated by EXO‐PD‐L1‐HGF treatment were enriched in the pathways of neurogenesis, regulation of cell development, positive regulation of cell differentiation, and chemokine signaling. e) Stat3 and Foxo3 were significantly expressed in the four significantly enriched gene sets. f) Gene concept network analysis of the significantly differentially expressed genes associated with the four enriched functions detailed that both Stat3 and Foxo3 were linked to the GSEA‐enriched functional terms. g,h) Validation of the mRNA expression of STAT3 and FOXO3 revealed a significant increase upon EXO‐PD‐L1‐HGF treatment in NPCs. Data are presented as mean ± SD (*p < 0.05; **p < 0.01). GO, Gene Ontology; GSEA, Gene Set Enrichment Analysis; KEGG, Kyoto Encyclopedia of Genes and Genomes; NPCs, neural progenitor cells; PCCs, primary cortical cells.

**Figure 5:**
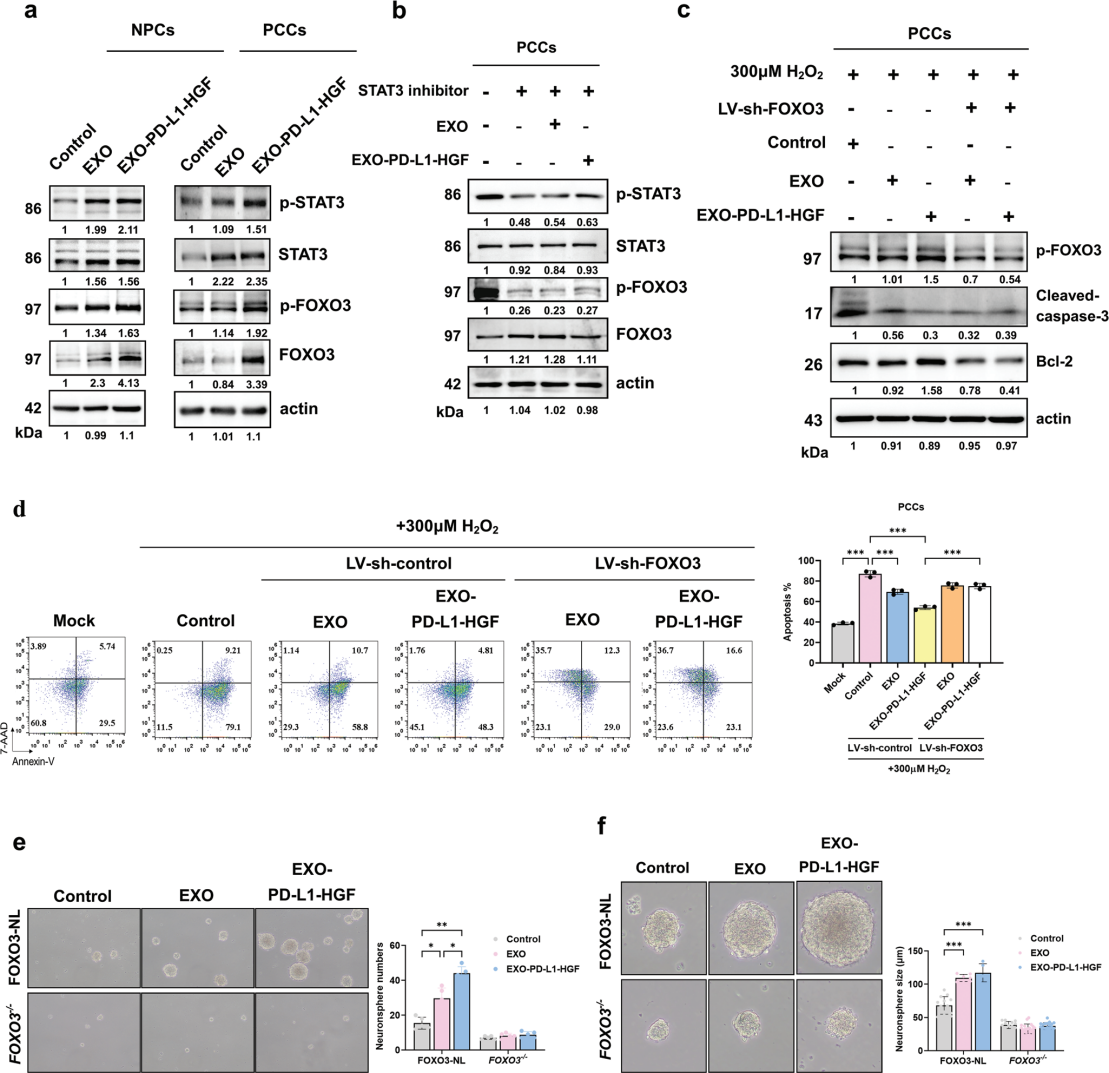
EXO‐PD‐L1‐HGF positively regulated the STAT3/FOXO3 pathway involved in neuroprotection and neuroregeneration. a) Expression of phospho‐STAT3 and phospho‐FOXO3 in NPCs and PCCs upon control, EXO, and EXO‐PD‐L1‐HGF treatments. Actin was used as the loading control. b) The protein expression levels of phospho‐STAT3 and phospho‐FOXO3 in PCCs were decreased upon addition of a stat3 inhibitor, as observed in the western blot. c) The western blot result showed that knockdown of FOXO3 expression reduced the increased levels of phospho‐FOXO3 and Bcl‐2 protein expression induced by EXO‐PD‐L1‐HGF, and enhanced the suppression of cleaved caspase‐3 levels upon treatment with 300 µm H_2_O_2_. d) In vitro apoptosis results showed that knockdown of FOXO3 expression suppressed the attenuation of cell death induced by EXO‐PD‐L1‐HGF upon treatment with 300 µm H_2_O_2_. e) The number of neurospheres formed by *FOXO3^‐/‐^* mice pretreated with control, EXO, and EXO‐PD‐L1‐HGF was determined. *FOXO3^‐/‐^* repressed the promotion of neurosphere formation by EXO‐PD‐L1‐HGF. f) The size of neurospheres formed by *FOXO3^‐/‐^* mice pretreated with control, EXO, and EXO‐PD‐L1‐HGF was measured. *FOXO3^‐/‐^* mice suppressed the increase in size promoted by EXO‐PD‐L1‐HGF. Quantified data are shown next to each graph. Data are presented as mean ± SD (*p < 0.05; **p < 0.01).

Next, we elucidated the mechanism underlying the participation of EXO‐PD‐L1‐HGF in the NPC‐enhanced neurotrophic effects. For this, we performed RNA‐based next‐generation sequencing to analyze the total RNA transcript expression in NPCs treated with control, EXO, and EXO‐PD‐L1‐HGF. Multivariate analysis using clustering heatmaps showed that several neurogenesis‐related genes, including HGF receptor, vascular endothelial growth factor (VEGF), bFGF, CXCR4, STAT3, and FOXO3, displayed not only a slight upregulation in the EXO‐treated group, but also a significant increase in expression in the EXO‐PD‐L1‐HGF‐treated group (**Figure** **4**a). Upon comparing the expression profiles of cells in the EXO‐PD‐L1‐HGF‐treated and control groups, we identified 311 upregulated genes after EXO‐PD‐L1‐HGF treatment, using fold‐change>1.5 and an adjusted *p*‐value < 0.05 as the threshold (Figure 4b). Gene ontology (GO) annotation revealed that these differentially expressing genes were involved in functions related to cell development, cell differentiation, response to stimuli, and cell motility (Figure 4c). Consistent with this, another functional enrichment analysis, gene set enrichment analysis (GSEA), using both the GO and Kyoto Encyclopedia of Genes and Genomes pathway databases, indicated that genes mediated by EXO‐PD‐L1‐HGF treatment were associated with four gene sets: neurogenesis (GO: 0022008), regulation of cell development (GO: 0060284), positive regulation of cell differentiation (GO: 0045597), and chemokine signaling pathway (adjusted *p* < 0.01). Most genes upregulated in the EXO‐PD‐L1‐HGF‐treated group, as compared with that in the EXO‐treated group were mapped to the leading edge subsets of the associated significant gene sets (Figure 4d). To understand the critical roles commonly involved in enriched functions, we subsequently identified the overlapping genes between each functional term enriched using GSEA. Only two genes were shared among the four significantly enriched gene sets (Figure 4e). Moreover, gene concept network analysis of the significantly differentially expressed genes associated with the four enriched functions showed that both Foxo3 and Stat3 were linked to the GSEA‐enriched functional terms (Figure 4f). Furthermore, validation of the mRNA expression of STAT3 and FOXO3 indicated a significant increase following EXO‐PD‐L1‐HGF treatment of the NPCs (Figure 4g,h). In addition, there was a significant increase in the protein expression levels of phospho‐STAT3 and phospho‐FOXO3 upon EXO‐PD‐L1‐HGF treatment of NPCs and PCCs (**Figures** **5**a and S5, Supporting Information). Collectively, these results suggested that Foxo3 and Stat3 may play important roles as transcription factors in the response to EXO‐PD‐L1‐HGF treatment.

Subsequently, to investigate whether EXO‐PD‐L1‐HGF regulates neuroplasticity via the STAT3‐FOXO3 signaling pathway, we pretreated PCCs with 20 µm of STAT3 inhibitor for 6 h and then subjected the cells to control, EXO, and EXO‐PD‐L1‐HGF treatments. Notably, western blot analysis showed that the inhibition of phospho‐STAT3 suppressed EXO‐PD‐L1‐HGF‐induced phospho‐FOXO3 expression in PCCs (Figure 5b and Figure S6, Supporting Information). To further confirm whether the transcription factor FOXO3 plays a significant role in neuroprotection and neuroregeneration, we knocked down FOXO3, which significantly reduced the EXO‐PD‐L1‐HGF‐attenuated H_2_O_2_‐induced neuronal apoptosis in PCCs, as demonstrated using western blot (Figure 5c and Figure S7, Supporting Information) and Annexin V‐FITC/7‐AAD double staining (Figure 5d). Moreover, knockout of FOXO3 attenuated the EXO‐PD‐L1‐HGF‐promoted neurosphere formation (Figure 5e,f). Taken together, these results suggested that the EXO‐PD‐L1‐HGF‐induced activation of the STAT3/FOXO3 cascade has the potential to suppress detrimental injury and rescue neural damage.

### 2.6 EXO‐PD‐L1‐HGF Contained Various Anti‐inflammatory Cytokines and Trophic Factors, as Assessed Using a Cytokine Array

To systemically investigate the modulating molecules encapsulated in the exosomes, we performed human cytokine arrays to analyze the important factors present in EXO and EXO‐PD‐L1‐HGF. Since MSCs can exert immunomodulatory effects through paracrine secretion, such as that of IL‐10 and transforming growth factor β‐(TGFβ),^[^ ^18^ ^]^ these exosomes were subjected to cytokine analyses. Upon comparing the two exosomes, we observed elevated levels of cytokines, including leukemia inhibitory factor (LIF), brain‐derived neurotrophic factor (BDNF), VEGF, fibroblast growth factor 2 (FGF2), and HGF, in EXO‐PD‐L1‐HGF than in EXO (Figure S8, Supporting Information).

### 2.7 EXO‐PD‐L1‐HGF Is Prominently Targeted into the Ischemic Brain

**Figure 6:**
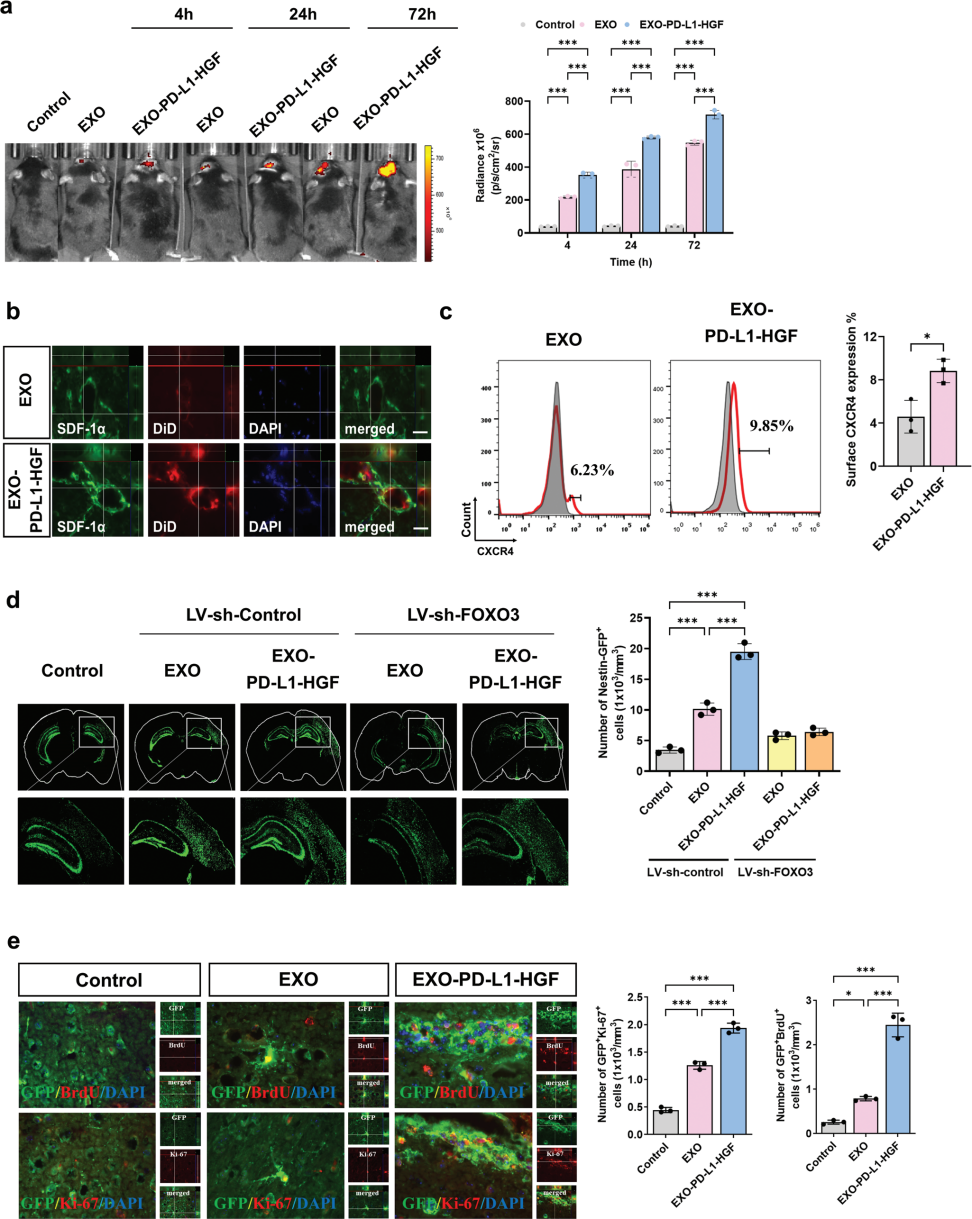
EXO and EXO‐PD‐L1‐HGF prominently migrated into the ischemic brain, owing to the upregulated expression of CXCR4. a) Control, DiD‐EXO, and DiD‐EXO‐PD‐L1‐HGF were intravenously injected into ischemic stroke mice. The fluorescence intensity of EXO‐PD‐L1‐HGF was observed at 4, 24, and 72 h after injection, using an IVIS system. b) Immunofluorescence showed that SDF‐1α was expressed in the ischemic injured area, and DiD‐EXO‐PD‐L1‐HGF migrated significantly to the injured site. c) Surface expression of CXCR4 was higher in the EXO‐PD‐L1‐HGF and EXO groups. d) Knockdown of FOXO3 expression suppressed the EXO‐PD‐L1‐HGF‐mediated promotion of the migration of nestin‐GFP‐expressing NPCs to the injured site. e) BrdU and Ki‐67 were used as cell proliferation markers to observe the effect of EXO‐PD‐L1‐HGF treatment on the proliferation of nestin‐GFP‐expressing cells. Quantified data are shown next to each graph. Data are presented as mean ± SD (*p < 0.05; **p < 0.01). PD‐L1, programmed death‐ligand 1; HGF, hepatocyte growth factor; GFP, green fluorescent protein; SDF‐1α, stromal cell‐derived factor‐1 alpha.

To examine the biodistribution of DiD‐labeled exosomes in vivo, ischemic stroke mice were analyzed at 4, 24, and 72 h after DiD‐EXO‐PD‐L1‐HGF and DiD‐EXO injection into mice via the femoral vein, using the IVIS System. The fluorescence signal of each exosome in the in vivo imaging system (IVIS) was detectable at 4 h after injection, followed by an obvious increase in fluorescence intensity at the stroke site. The DiD‐EXO‐PD‐L1‐HGF‐treated stroke brain displayed a significant time‐dependent enhancement of the fluorescent signal than the EXO‐treated and control brains (**Figure** **6**a). Consistently, more stromal cell‐derived factor‐1 alpha (SDF‐1α)‐expressing cells co‐localized with DiD‐labeled exosomes in the DiD‐EXO‐PD‐L1‐HGF‐treated groups than that in the DiD‐EXO‐treated and control groups, which indicated the homing ability of EXO‐PD‐L1‐HGF under the SDF‐1α gradient poststroke (Figure 6b). Since the ischemic stroke enhanced the SDF‐1α expression mainly in the vascular tissue,^[^ ^19^ ^]^ that showed the co‐localization of SDF‐1α with Laminin^+^ vascular tissue in Figure S9 in the Supporting Information. The SDF‐1α/CXCR4 axis plays a crucial role in the homing of MSCs to the sites of injury.^[^ ^20^ ^]^ To investigate whether the homing effect of EXO‐PD‐L1‐HGF was enhanced with increasing CXCR4 expression, we analyzed the surface CXCR4 expression of EXO and EXO‐PD‐L1‐HGF using flow cytometry, which indicated significantly higher expression in the EXO‐PD‐L1‐HGF‐treated group than that in the EXO‐treated group (Figure 6c). Moreover, immunofluorescence analysis showed that after intravenous injection into C57BL/6 mice at 72 h postischemic stroke, the red fluorescent DiD‐EXO‐PD‐L1‐HGF was co‐localized with glial fibrillary acidic protein (GFAP)‐ or MAP2‐expressing (green fluorescence) cells in the brain with stroke (Figure S10, Supporting Information). Taken together, these data indicated that systemically implanted EXO‐PD‐L1‐HGF and EXO were capable of accumulating in the ischemic brain and then endocytosing into neuroglial cells to exert their regenerative effect.

### 2.8 Intravenous Injection of EXO‐PD‐L1‐HGF Augmented the Endogenous Nestin‐ GFP^+^ Cell‐Induced Neurogenesis in the Peri‐Infarct Area

To gain a better understanding of the regenerative potential of endogenous nestin‐ green fluorescent protein (GFP)‐expressing cells after each treatment protocol, we used nestin‐GFP transgenic mice^[^ ^21^ ^]^ to investigate the migration and proliferation of nestin‐GFP‐expressing cells in the sensorimotor cortex poststroke. At 7 d poststroke, the EXO‐PD‐L1‐HGF‐treated group displayed a significant increase in the number of nestin‐GFP‐expressing cells that migrated toward the lateral and ventral peri‐infarct areas than the EXO‐treated and control groups. In contrast, knockdown of FOXO3 expression repressed the EXO‐PD‐L1‐HGF‐promoted nestin‐GFP‐expressing cell migration in the infarct areas (Figure 6d). Moreover, EXO‐PD‐L1‐HGF administration significantly increased the number of GFP^+^BrdU^+^ and GFP^+^Ki‐67^+^ cells (Figure 6e).

**Figure 7:**
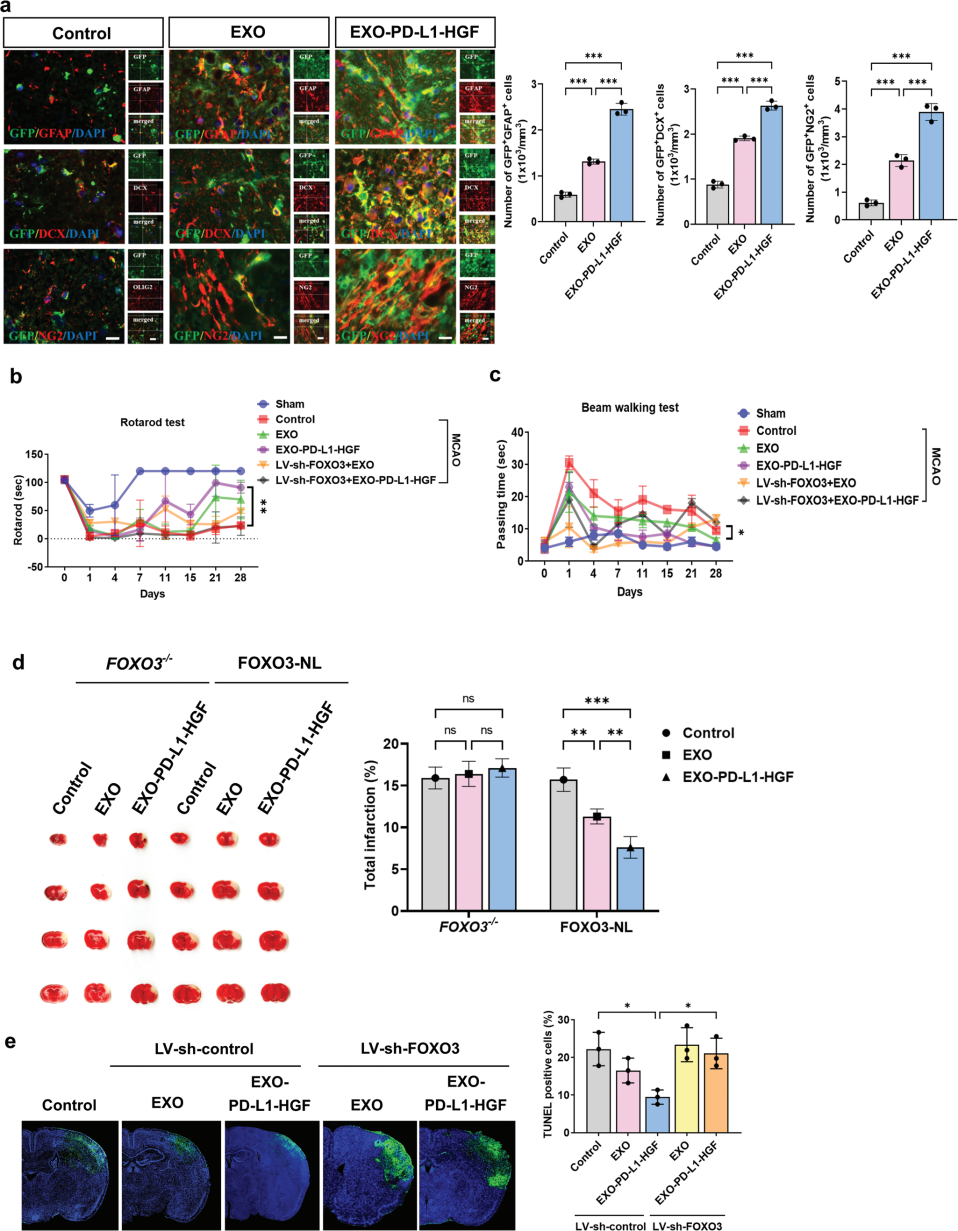
Activation of FOXO3 modulated the EXO‐PD‐L1‐HGF‐enhanced functional recovery and infarct volume reduction poststroke. a) Immunofluorescence showed that EXO‐PD‐L1‐HGF treatment promoted the differentiation of NPCs. NG2 is an oligodendrocyte marker, while GFAP is an astrocyte marker, and DCX is an immature neuron marker. b) Rotarod and c) beam walking tests result showed that knockout of FOXO3 expression (*FOXO3* ^*–/–*^) suppressed the neurological function improvement induced by EXO‐PD‐L1‐HGF. d) TTC staining results showed that *FOXO3* ^*–/–*^ mice repressed the attenuation of infarction size over the ischemia hemisphere. The quantified data are shown next to each graph. e) TUNEL staining was performed to detect apoptosis in the ischemia region. Cell nuclei were counterstained with DAPI (blue). Quantitative estimation of the proportion of apoptotic cells in each experimental group: LV‐sh‐control and LV‐shFOXO3 pretreatment followed by treatment with control, EXO, and EXO‐PD‐L1‐HGF, as indicated. Data are presented as mean ± SD (*p < 0.05; **p < 0.01). FOXO3, forkhead box 3; TTC, triphenyltetrazolium chloride; PD‐L1, programmed death‐ligand 1; HGF, hepatocyte growth factor; TUNEL, terminal deoxynucleotidyl transferase dUTP nick‐end labeling; DAPI, 4′,6‐diamidino‐2‐phenylindole; NPCs, neural progenitor cells; GFAP, glial fibrillary acidic protein; NG2, neural/glial antigen 2; DCX, doublecortin.

A previous study demonstrated that ischemic stroke stimulates the proliferation of endogenous NPCs that ectopically migrate to the infarct area.^[^ ^22^ ^]^ Moreover, the functional integration of proliferating NPCs with subsequent adult neurogenesis enhances stroke recovery.^[^ ^23^ ^]^ To investigate whether EXO‐PD‐L1‐HGF promotes the proliferation of endogenous NPCs to induce neuroglial differentiation and adult neurogenesis for functional recovery, co‐localization of neuroglial and proliferation markers with nestin‐GFP‐expressing populations was assessed by means of immunohistochemistry in nestin‐GFP mice poststroke. In addition to nestin‐GFP‐expressing oligodendrocytes (GFP^+^NG2^+^) and astrocytes (GFP^+^GFAP^+^), we also focused on the expression of doublecortin (DCX) markers in nestin‐GFP^+^ populations. Importantly, the EXO‐PD‐L1‐HGF‐treated group displayed more GFP^+^DCX^+^, GFP^+^NG2^+^, and GFP^+^GFAP^+^ cells within the ventral peri‐infarct region at 1 week poststroke than the EXO‐treated and control groups (**Figure** **7**a).

### 2.9 Activation of FOXO3 Signaling Modulated the EXO‐PD‐L1‐HGF‐Enhanced Functional Recovery and Infarct Volume Reduction Poststroke

To verify the neuroregenerative ability of EXO‐PD‐L1‐HGF through the activation of the FOXO3 pathway in mice with stroke, we performed infarct volume measurements by means of triphenyltetrazolium chloride (TTC) staining and neurological functional assessments of control, EXO (with FOXO3‐NL or *FOXO3^–/–^*)‐treated, and EXO‐PD‐L1‐HGF (with FOXO3‐NL or *FOXO3^–/–^*)‐treated ischemic stroke models. Compared with the other groups, the EXO‐PD‐L1‐HGF treatment group displayed a significant improvement in neurobehavioral examination, as measured by means of rotarod treadmill testing and beam walking, at 28 d postischemic stroke. In contrast, stereotaxic administration of LV‐shFOXO3 to the EXO‐ and EXO‐PD‐L1‐HGF‐treated groups significantly suppressed the EXO‐PD‐L1‐HGF‐induced improvement in neurological function (Figure 7b,c). Consistent with this finding, TTC staining showed that the infarction size over the ischemic hemisphere was smaller in the EXO‐PD‐L1‐HGF‐treated group than in the EXO‐treated and control groups. However, the EXO‐PD‐L1‐HGF‐induced reduction in infarction volume was not observed in the *FOXO3^–/–^* mice (Figure 7d). Furthermore, there was a significant reduction in the number of TUNEL‐positive cells in the ischemic hemisphere treated with EXO‐PD‐L1‐HGF, whereas treatment with LV‐shFOXO3 abrogated this EXO‐PD‐L1‐HGF‐induced decrease (Figure 7e). In summary, these results suggested that FOXO3 activation switches on the EXO‐PD‐L1‐HGF‐modulated neuroplasticity in the stroke brain.

### 2.10 Intravenous Injection of EXO‐PD‐L1‐HGF Reduces the Proinflammatory Responses via Regulation of Anti‐inflammatory Leukocytes in the Ischemic Brain and Spleen

**Figure 8:**
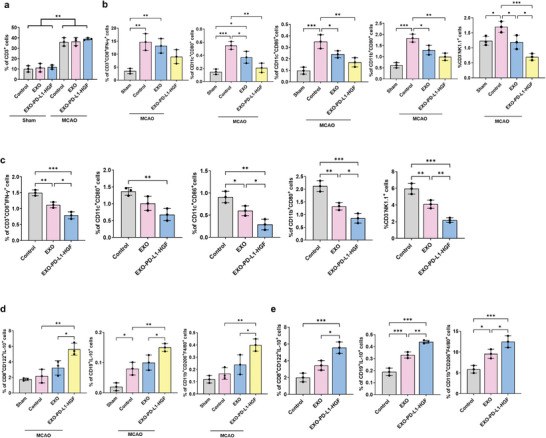
Intravenous injection of EXO‐PD‐L1‐HGF reduces proinflammatory responses via regulation of anti‐inflammatory leukocytes in the ischemic brain and spleen. a) The number of CD3‐expressing T cells was higher in the ischemic hemisphere, as compared to that in either hemisphere. In the b) ischemic hemisphere and c) spleen, there was a significantly reduced percentage of CD11c^+^CD80^+^ and CD11c^+^CD86^+^ (activated dendritic cells), CD3^+^CD8^+^IFN‐γ^+^ (cytotoxic T cells), CD3^–^NK1.1^+^ (NK cells), and CD11b^+^CD80^+^ cells (M1 macrophage cells) in the EXO‐PD‐L1‐HGF treatment group. In the d) ischemic hemisphere and e) spleen, there was an enhanced percentage of CD‐19^+^IL‐10^+^ (regulatory B cells), CD8^+^CD122^+^IL‐10^+^ (Treg cells), and CD11b^+^CD206^+^F4/80^+^ cells (M2 macrophage cells) in the EXO‐PD‐L1‐HGF treatment group. Data are presented as mean ± SD (*p < 0.05; **p < 0.01). PD‐L1, programmed death‐ligand 1; HGF, hepatocyte growth factor; CD, cluster of differentiation; IFN‐γ, interferon gamma.

To determine whether EXO‐PD‐L1‐HGF modulates immunoregulation, the percentage of leukocytes in the brain and spleen after stroke was examined using flow cytometry. The gating strategy followed for flow cytometry is depicted as a uniform procedure in Figure S11a in the Supporting Information. The ischemic hemisphere treated with EXO‐PD‐L1‐HGF showed a significant increase in the total number of viable leukocytes, including CD3^+^ T cells, as compared with the sham group, whereas the total cell numbers did not change between the treatment and control groups in either hemisphere (**Figure** **8**a). EXO‐PD‐L1‐HGF treatment caused a significant increase in the size of the spleen than the EXO and control treatments (Figure S11b, Supporting Information). Intravenous administration of EXO‐PD‐L1‐HGF to the mice with stroke significantly reduced the percentages of CD11c^+^CD80^+^ and CD11c^+^CD86^+^ (activated dendritic cells), CD3^+^CD8^+^IFN‐γ^+^ (cytotoxic T cells), CD3^–^NK1.1^+^ (natural killer cells), and CD11b^+^CD80^+^ cells (M1 macrophage cells) (Figure 8b,c), but enhanced the percentages of CD19^+^IL‐10^+^ (regulatory B cells), CD8^+^CD122^+^IL‐10^+^ (Treg cells), and CD11b^+^CD206^+^F4/80^+^ cells (M2 macrophage cells) (Figure 8d,e) in the ischemic hemisphere and spleen, as compared with that in the EXO‐treated and control groups.

## 3 Discussion

Owing to their biological functions of multilineage differentiation, tissue repair promotion, immunomodulation, and neuroprotection, MSCs serve as promising candidates for the treatment of stroke. However, it is still challenging to overcome some unresolved issues in the clinical applications of MSCs. With respect to the mechanisms underlying these biological functions, it was originally thought that MSCs home to injured tissues, differentiate, and replace damaged cells. Moreover, trophic factors, including VEGF, HGF, and insulin‐like growth factor‐1, play important roles in the homing and survival of MSCs. HGF is a pleiotropic growth factor of mesenchymal origin that enhances the motility, proliferation, migration, and survival of MSCs. A previous study showed that high levels of HGF induced the homing of bone marrow mesenchymal stem cells (BMSCs)to the injured tissue by increasing the expression of miRNA‐221 and −26b through the activation of the AKT and focal adhesion kinase pathways.^[^ ^24^ ^]^ One study showed that priming BMSCs with HGF enhanced vascular regeneration and restored cardiac function during myocardial infarction.^[^ ^25^ ^]^

PD‐L1 is expressed on MSCs^[^ ^26^ ^]^ and the PD‐1/PD‐L1 pathway mediates the immunosuppressive mechanism of MSCs.^[^ ^27^ ^]^ Moreover, a previous study indicated massive production of inflammatory factors poststroke, transmigration of splenocytes to the circulation, and infiltration of ischemic areas of the brain by T cells, macrophages, and inflammatory polymorphonuclear cells, resulting in a worse stroke outcome. Notably, PD‐L1 could control inflammation after ischemic stroke^[^ ^28^ ^]^ and significantly attenuate neurological deficits, ameliorate the inflammatory environment, and enhance the blood–brain barrier integrity in mice with intracerebral hemorrhage, by repressing the mammalian target of rapamycin pathway.^[^ ^28^, ^29^ ^]^ Therefore, we genetically engineered MSCs overexpressing HGF and PD‐L1 to improve homing to target sites, modulate immunoreactions, and repair damaged tissues. However, the engraftment with MSCs at injury sites is sparse and transient.^[^ ^30^ ^]^ Several studies have also indicated a poor survival rate when MSCs enter a hypoxic environment, and neuroglial cells are injured by inflammatory cytokines poststroke, which impedes the therapeutic efficacy of MSCs.^[^ ^31^ ^]^

Currently, MSC‐derived exosomes are considered novel therapeutic tools for cell‐to‐cell communication. Moreover, several studies have indicated that MSC‐derived exosomes exert their effects through the horizontal transfer of proteins, nucleic acids, regulatory miRNAs, and long noncoding RNAs, leading to functions similar to those of MSCs, such as a reduction in the size of myocardial infarctions, facilitation of brain injury repair, and modulation of immune responses.^[^ ^32^ ^]^ Additionally, several studies have suggested that engineered exosomes could further enhance their capacity by modulating parental cells.^[^ ^33^ ^]^ One study suggested that ADSC‐derived exosomes enriched with miR‐30d‐5p can protect against acute ischemic stroke by preventing cerebral injury through the inhibition of autophagy‐mediated microglial polarization to M1.^[^ ^34^ ^]^ Another study demonstrated that intravenous administration of ADSC‐derived exosomes enriched with miR‐126 improves functional recovery, enhances neurogenesis, and inhibits neuroinflammation after stroke.^[^ ^35^ ^]^ Moreover, research showed that exosomes from Akt‐overexpressing MSCs enhance cardioprotection and angiogenesis in myocardial infarction therapy, with platelet‐derived growth factor D playing a crucial role in this process.^[^ ^36^ ^]^ To address the unresolved issues mentioned above, this study aimed to develop a strategy to enhance the therapeutic efficacy of genetically engineered MSC‐derived exosomes, namely, EXO‐PD‐L1‐HGF.

Poststroke neurogenesis has received increasing attention in recent decades and is a promising therapeutic strategy for ischemic stroke. Ischemic stroke is a common cerebrovascular accident that occurs in adults and poses a high risk of mortality. Currently, ischemic stroke is treated by administering a thrombolytic agent, such as a tissue plasminogen activator, or by performing a surgical thrombectomy to mechanically remove blood clots. However, many patients with ischemic stroke face challenges in receiving such therapies because of delays in hospital admission. Therefore, our study focused on the contribution of EXO‐PD‐L1‐HGF to the repair of damaged areas and improvement in neural function after ischemic stroke.

We observed that ADSC‐derived EXO‐PD‐L1‐HGF promoted the antiapoptotic capability of neural cells, enhanced the proliferative ability of endogenous NPCs, and modulated their immunosuppressive capability via the upregulation of CD8^+^CD122^+^IL‐10^+^ Tregs, leading to improved outcomes poststroke. Verification of the mechanism of EXO‐PD‐L1‐HGF in the stroke model by means of RNA‐sequencing analysis revealed upregulation of FOXO3 mRNA expression in the EXO‐PD‐L1‐HGF‐treated group, verified the EXO‐PD‐L1‐HGF‐induced proliferation of NPCs, and recognized the involvement of the STAT3/FOXO3 pathway in the neuroprotective effects after MCA occlusion injury. Moreover, FOXO3 acts as a critical downstream regulator of the phosphoinositide 3‐kinase/AKT pathway and regulates numerous genes involved in various cellular processes such as proliferation, survival, metabolism, differentiation, and the cell cycle.^[^ ^14^ ^]^ Stroke‐induced NPC cell proliferation, migration, differentiation, and survival are associated with spontaneous functional recovery.^[^ ^37^ ^]^ However, the role of endogenous NPCs in brain self‐repair and spontaneous functional recovery after a stroke remains unclear. We observed that EXO‐PD‐L1‐HGF promoted NPC proliferation in both the SVZ and subgranular zone during the 4 d following ischemic stroke. Additionally, EXO‐PD‐L1‐HGF induced a paracrine effect on NPC‐released trophic factors, such as brain‐derived neurotrophic factor, VEGF, and glial cell line‐derived neurotrophic factor, which may contribute to neural network remodeling and functional recovery.

## 4 Conclusions

In summary, our results showed that EXO‐PD‐L1‐HGF is an efficient tool for gene drug delivery to the ischemic area and a potential product for novel cell‐free therapy in regenerative medicine.

## 5 Experimental Section

### Cell Culture

The cell line of the human telomerase reverse transcriptase‐immortalized adipose tissue‐derived MSCs (hTERT‐ADSCs or ASC52telo; Cat# SCRC‐4000) was purchased from American Type Culture Collection (ATCC). The certificate of analysis for the hTERT‐ADSC cell line provided by ATCC indicated that the immortalized cells still exhibited the characteristics of MSCs, including high expression of CD73, CD90, and CD105, but lacked the expression of CD45, CD34, CD19, CD11b, and human leukocyte antigen‐DR isotype. The hTERT‐ADSC cells were maintained with MSC *NutriStem XF* Medium and *Supplement Mix (Sartorius, USA*). Moreover, hTERT‐ADSCs differentiated into osteoblasts, chondrocytes, and adipocytes in vitro. Human SH‐SY5Y cells were cultured in Dulbecco's modified Eagle medium/F12 (1:1, v/v) (Cat# 11330032, Gibco, USA) supplemented with 10% fetal bovine serum (FBS) (Cat# SH30084.03, Cytiva, USA) and 1% penicillin‐streptomycin (Cat# 15140122, Gibco, USA). The cells were grown in a humidified atmosphere with 5% CO_2_ at 37 °C.

### Preparation, Isolation, and Characterization of Primary Adipose Tissue‐Derived MSCs (ADSCs)

The collection of human adipose tissues was approved by the Institutional Review Board of China Medical University Hospital. The tissues were washed three times with Ca^2+^‐ and Mg^2+^‐free phosphate‐buffered saline (PBS) (Thermo Fisher Scientific, USA), then mechanically dissociated and extensively cut into pieces smaller than 0.5 cm^3^ using scissors, treated with collagenase type 1 (Sigma‐Aldrich, USA), and incubated for 3 h at 37 °C, in a humidified atmosphere with 5% CO_2_. The explants were cultured in MSC *NutriStem XF* Medium with *Supplement Mix* and antibiotics, at 37 °C, in a humidified atmosphere with 5% CO_2_. The cells were left undisturbed for 5–7 d to allow their migration from the explants. The cellular morphology of the ADSCs became homogenously spindle‐shaped in the cultures after 4–8 passages, following which the specific surface molecules of the cells were characterized by means of flow cytometry. The cells were detached with TrypLE Select Enzyme (Cat# 12563029, Gibco, USA), washed with PBS, and incubated with the respective antibodies conjugated with FITC or phycoerythrin, including those against CD13, CD29, CD44, CD73, CD90, CD105, CD166, CD49b, CD1q, CD3, CD10, CD14, CD31, CD34, CD45, CD49d, CD56, CD117, and human leukocyte antigen‐ABC and DR isotypes (BD Pharmingen, USA). Thereafter, the cells were analyzed using an Attune NxT Flow cytometer (Thermo Fisher Scientific, USA) with NxT software and FlowJo software v.7.6.

### Generation of PD‐L1‐HGF‐Overexpressing Cells Using the PiggyBac Transposon System

Human PD‐L1‐ and HGF‐tagged open reading frame clones (Cat# RC213071 and Cat# RC215593L3, respectively; OriGene Technologies, USA) were used as the polymerase chain reaction (PCR) templates for generating the PD‐L1 and HGF complementary DNA (cDNA), respectively. The bicistronic expression construct was generated by inserting the PD‐L1 and HGF cDNA fragments into pSF‐CMV‐CMV‐SbfI (Oxford Genetics, UK) using a specific restriction enzyme linker (T2A sequence), to obtain the pSF‐PD‐L1‐T2A‐HGF plasmid. Subsequently, the PD‐L1‐T2A‐HGF sequence was PCR‐amplified and sub‐cloned into the PB‐CMV‐MCS‐EF1α‐Puro expression vector (Cat# PB510B, System Biosciences, USA) using NotI, to generate a PB510B‐PD‐L1‐T2A‐HGF expression construct. To generate hTERT‐ADSC‐PD‐L1‐HGF cells, the PB510B‐PD‐L1‐T2A‐HGF plasmid was cotransfected with PiggyBac transposase (System Biosciences, USA) into hTERT‐ADSCs, using an Amaxa Nucleofector 2b device (Lonza, Switzerland). The experimental procedure was conducted according to the protocol provided by the manufacturer. In brief, the cells (2 × 10^5^ cells) were harvested and resuspended in an appropriate electroporation buffer and the cell suspension was combined with the DNA solution in an electroporation cuvette before applying a brief electrical pulse. And, the stable cells were selected using puromycin (2 µg/µL).

### In Vitro Differentiation Assays

For adipogenic differentiation, confluent monolayer cultures of primary ADSCs, hTERT‐ADSCs, and hTERT‐ADSC‐PD‐L1‐HGF were grown in an adipogenic differentiation medium consisting of StemPro adipogenic differentiation basal medium (Cat# A10410–01, Gibco, USA), supplement (Cat# A10065‐01, Gibco, USA), 15% rabbit serum, and 1% penicillin‐streptomycin. The cells that were maintained in the ordinary MSC NutriStem XF Medium served as negative controls. The adipogenic medium was changed three times per week, for 7–14 d. To assess adipogenic differentiation in terms of intracellular lipid accumulation, the cells were stained with 0.3% Oil Red staining solution (Sigma‐Aldrich, USA) for 30 min at room temperature, while preventing exposure to light. For osteogenic differentiation, confluent monolayer primary ADSC, hTERT‐ADSC, and hTERT‐ADSC‐PD‐L1‐HGF cultures were grown in osteogenic medium containing osteocyte/chondrocyte differentiation basal medium (Cat# A10069‐01, Gibco, USA), supplement (Cat# A10066‐01, Gibco, USA), and 1% penicillin‐streptomycin (Cat# 15140122, Gibco, USA). The osteogenic medium was changed three times per week for 21–28 d. To detect calcium mineralization, the levels of osteogenesis were determined using 2% Alizarin Red S staining solution (Sigma‐Aldrich, USA) for 45 min at room temperature, while preventing exposure to light. For chondrogenic differentiation, confluent monolayer primary ADSC, hTERT‐ADSC, and hTERT‐ADSC‐PD‐L1‐HGF cultures were grown in chondrogenic medium consisting of osteocyte/chondrocyte differentiation basal medium, supplement (Cat# A10064‐01, Gibco, USA), and 1% penicillin‐streptomycin. The chondrogenic medium was changed twice per week for 14–21 d. Chondrocyte spheres were confirmed histologically by carrying out Alcian blue staining (Sigma‐Aldrich, USA) of sulfated proteoglycans overnight at room temperature, while preventing exposure to light.

### Purification of Exosomes

hTERT‐ADSCs and hTERT‐ADSC‐PD‐L1‐HGF cells were cultured conventionally in MSC NutriStem XF Medium with 1% penicillin‐streptomycin in 75‐cm^2^ tissue culture flasks. For exosome generation, the conventional culture medium for hTERT‐ADSCs (EXO) and hTERT‐ADSC‐PD‐L1‐HGF (EXO‐PD‐L1‐HGF) was replaced with fresh Dulbecco's modified Eagle medium (Cat# 11 965 092, Gibco, USA) without FBS and the conditioned medium was collected. For exosome isolation, the cell culture supernatants were centrifuged at 500 × *g* for 10 min and further centrifuged at 3000 × *g* for 20 min, to eliminate cells and debris. The clarified supernatant was concentrated using Amicon Ultra‐15 Centrifugal Filter 10K Devices (Cat# UFC901024, Merck Millipore, USA) and then centrifuged at 5000 × *g* for 60 min at 4 °C before being filtered using 0.22‐µm pore filters. The concentrated supernatants were incubated with Total Exosome Isolation reagent (Cat# 4 478 359, Thermo Fisher Scientific, USA) overnight at 4 °C and then harvested by means of centrifugation at 10,000 × *g* for 60 min at 4 °C. Exosomes were used freshly or stored at −80 °C after resuspension in sterile 1× PBS.

### Transmission Electron Microscopy

The sample solution (exosomes) was dropped onto a carbon‐coated Cu grid. The droplet residues were removed and the copper grid was dried in a vacuum desiccator at room temperature. Electron microscopy images were captured and analyzed using a transmission electron microscope (JSM‐2100, JEOL, Japan) operating at 80 kV.

### Nanoparticle Tracking Analysis

The exosomes were diluted in sterile 1× PBS to a final volume of 1 mL. The size and concentration of the exosomes isolated from the cell culture supernatants were determined using a ZetaView MONO nanoparticle tracking analysis system (Particle Metrix, Germany) equipped with fast video capture and particle‐tracking software (ZetaView Software 8.04.02 SP2).

### Western Blot Analysis

Immunoblotting was performed as described previously.^[^ ^38^ ^]^ Whole‐cell lysates or exosomal proteins were separated by means of 8–10% sodium dodecyl sulfate‐polyacrylamide gel electrophoresis and transferred onto nitrocellulose membranes. The blots were blocked with 5% nonfat dry milk at room temperature for 60 min and incubated overnight at 4 °C with the corresponding primary antibodies (at dilutions recommended by the suppliers), followed by incubation with horseradish peroxidase‐conjugated secondary antibodies at room temperature for 60 min. The membranes were developed with enhanced chemiluminescence (ECL) detection reagents (Thermo Fisher Scientific, USA). CD63 (clone: Ts63, Thermo Fisher Scientific, USA), CD9 (clone: ARC0330, ABclonal, USA), and CD81 (clone: ARC0615, ABclonal, USA) were used as exosomal markers. Antibodies against PD‐L1 from OriGene Technologies; HGF, phospho‐STAT3, STAT3, phospho‐FOXO3, and FOXO3 from Cell Signaling Technology; and CXCR4 from Novus Biologicals; and Bcl‐2, Bax, and cleaved caspase 3 from ABclonal were used to detect the respective proteins in the lysates. Actin (Sigma‐Aldrich, USA) was used as a loading control.

### Real‐Time Quantitative PCR

Total RNA was isolated from NPCs using TRIzol Reagent (Thermo Fisher Scientific, USA), and reverse transcribed into first‐strand cDNA with SuperScript III First‐Strand Synthesis SuperMix (Thermo Fisher Scientific, USA). Samples were analyzed using the QuantStudio Real‐Time PCR system (Thermo Fisher Scientific, USA). Glyceraldehyde 3‐phosphate dehydrogenase (GAPDH) was used as an internal control. Information regarding the primers used is provided in Table S1 in the Supporting Information.

### Enzyme‐Linked Immunosorbent Assay (ELISA)

For the detection of HGF, the supernatants of human hTERT‐ADSCs and hTERT‐ADSC‐PD‐L1‐HGF cells or exosomes were harvested and the levels in them were measured according to the manufacturer's instructions (Cat# DHG00B, R&D Systems, USA). For detection of IFN‐γ, the supernatants of human CD3^+^ T cells were harvested and the levels in them were measured according to the manufacturer's instructions (Cat# DIF50C, R&D Systems, USA).

### Flow Cytometry of Exosomal PD‐L1, CXCR4, CD63, CD9, and CD81

Exosomes were resuspended in sterile 1× PBS for further capture with streptavidin magnetic beads from the Exo‐Flow Capture Kit (Cat# EXOFLOW150A‐1, System Biosciences, USA) overnight at 4 °C. The beads were coupled with tetraspanin‐biotin antibodies (CD63, CD9, and CD81) from the Exo‐Flow Capture Kit, as well as biotin‐coupled anti‐PD‐L1 (clone: 29E.2A3) and anti‐CRCR4 (clone: 12G5) antibodies (both from BioLegend, USA). After washing away the unbound exosomes three times, the exosomes were further stained with Exo‐FITC for 2 h at 4 °C, in preparation for flow cytometry. The data generated on an Attune NxT Flow cytometer was used to set the gate settings and perform data analysis using FlowJo software v.7.6.

### Exosome Labeling Using Lipophilic Membrane Dyes In Vitro

Exosomes were labeled with 1 mg mL^−1^ 3,3′‐dioctadecyloxacarbocyanine, perchlorate (DiO) green dye (Thermo Fisher Scientific, USA) for 60 min at room temperature and repelleted using Total Exosome Isolation reagent overnight at 4 °C. After centrifugation at 10,000×*g* for 60 min at 4 °C, the DiO‐labeled exosomes were resuspended in sterile 1× PBS. After incubation of the DiO‐labeled exosomes with the DiD‐labeled cells for 60 min at 37 °C, images of exosome uptake were acquired with an SP2 Confocal Spectral Microscope.

### Isolation of Primary Cortical Cells (PCCs)

PCCs were prepared from Embryonic Day‐17 C57BL/6JNarl mice obtained from the National Laboratory Animal Center, Taiwan. Briefly, the isolated cerebral cortex was pooled, dissociated via mechanical trituration, and suspended in neurobasal medium with 2% B‐27 supplement, 0.5 mM l‐glutamine, 25 µM glutamic acid, and 1% penicillin‐streptomycin (all from Gibco, USA). Cells were counted by means of Trypan blue exclusion and plated on either poly‐d‐lysine‐coated plates (Nunc, USA), at a density of 3 × 10^4^ cells in a 24‐well plate or 2~3 × 10^6^ cells in a 6‐well plate. Cultures were maintained in a humidified incubator with 5% CO_2_ at 37 °C. After 3 d in vitro, half of the growth medium was removed and replaced with maintenance medium (neurobasal medium with 2% B‐27 supplement and 0.5 mm l‐glutamine). The maintenance medium was changed using the same method every 3–4 d. This method produced cultures in which more than 95% of the cells were neurons.

### In Vitro Cytotoxicity Assay

PCCs or SH‐SY5Y cells were seeded at a density of 3×10^5^ or 1×10^5^ in a 24‐well plate overnight and then treated with various concentrations of hydrogen peroxide (H_2_O_2_; 0, 100, 200, 300, 400, and 500 µM) for 24 h, in a humidified incubator with 5% CO_2_ at 37 °C. Subsequently, 300 µM H_2_O_2_ was selected for the treatment of the PCCs and SH‐SY5Y cells, to investigate whether the exosomes could exert a neuroprotective effect against H_2_O_2_‐induced cell death. PCCs or SH‐SY5Y cells were pretreated with EXO and EXO‐PD‐L1‐HGF (20 µg) overnight and then subjected to treatment with 300 µM H_2_O_2_ for 3 h, following which the cell viability was determined using CCK‐8 assay. The experimental procedure was conducted according to the CCK‐8 protocol provided by the manufacturer. In brief, 10 µL of CCK‐8 solution was added to each well containing 100 µL of culture medium, and the plate was incubated at 37 °C for 2 h. The absorbance was measured at 450 nm using a microplate reader.

### Terminal Deoxynucleotidyl Transferase dUTP Nick‐End Labeling (TUNEL) Assay

Cellular apoptosis was assessed by means of immunohistochemistry and immuno‐cytochemistry using a commercial TUNEL staining kit (DeadEnd Fluorometric TUNEL System, Promega, USA), as described previously.^[^ ^39^ ^]^ The percentage of TUNEL‐positive nuclei was calculated as the number of TUNEL‐positive nuclei divided by the total number of nuclei stained with 4′,6‐diamidino‐2‐phenylindole (DAPI).

### Annexin V Fluorescence Assay

The apoptotic effect on PCCs and SH‐SY5Y cells was studied using an Annexin V fluorescence kit (BD Biosciences, USA), according to the manufacturer's instructions. Briefly, the cells were seeded for 24 h, at a density of 2×10^6^ cells in a six‐well plate. Following that, the seeded cells were divided into three groups that were subjected to the respective pretreatments (control, EXO, and EXO‐PD‐L1‐HGF, at 20 µg each) overnight and then treated with 300 µM H_2_O_2_. Subsequently, the treated cells were harvested and collected as pellets. Next, the pellets were resuspended in 400 µL binding buffer and stained with 5 µL of FITC‐Annexin V and 5 µL of 7‐AAD provided in the kit. The cells were analyzed using an Attune NxT Flow Cytometer and FlowJo software v.7.6.

### T‐Cell Proliferation Assay

CD3^+^ T cells were isolated from peripheral blood mononuclear cells using magnetic‐activated cell sorting (MACS) human CD3 microbeads (Cat# 130‐050‐101, Miltenyi Biotec, Germany), and the purity of the CD3^+^ cell population was determined to be 97% by means of flow cytometry. The CD3^+^ T cells were then activated in KBM502 medium supplemented with 10% FBS and ImmunoCult Human CD3/CD28 T‐cell activator (Cat# 10971, STEMCELL Technologies, USA), at a ratio of 1:1 (25 µL of activator/1×10^6^ CD3^+^ T cells). These cells were then divided into three groups and subjected to the respective treatments (control, EXO, and EXO‐PD‐L1‐HGF, at 20 µg each). For the proliferation assay, activated CD3^+^ T cells were stained using the CFSE Cell Proliferation Kit (Cat# C34570, Thermo Fisher Scientific, USA). After 3 d of coculture, the activated T cells were harvested and incubated with an anti‐CD3 antibody (clone: OKT3, eBioscience, USA), at 4 °C for 60 min. Subsequently, the cells were analyzed using an Attune NxT Flow Cytometer (capturing 10000 events per sample) and FlowJo software v.7.6.

### Intracellular Cytokine Staining In Vitro

Intracellular cytokine staining was performed as previously described.^[^ ^40^ ^]^ In brief, CD3^+^ T cells were resuspended (1×10^6^ cells mL^−1^) and cultured with lipopolysaccharide (100 ng mL^−1^, Sigma), phorbol 12‐myristate 13‐acetate (50 ng mL^−1^, Sigma, USA), ionomycin (1 µg mL^−1^), and GolgiPlug protein transport inhibitor (BD Biosciences) for 16 h. The cells were fixed and permeabilized with a fixation/permeabilization buffer (BD Biosciences, USA), according to the manufacturer's instructions. The permeabilized cells were stained with an anti‐interleukin (IL)−10 antibody (clone: JES3‐19F1, BioLegend, USA), following which they were resuspended in staining buffer for image acquisition. Isotype‐matched monoclonal antibodies served as negative controls. The cells were analyzed using an Attune NxT Flow Cytometer and FlowJo software v.7.6.

### Isolation of NPCs and Neurosphere Self‐Renewal Assay

The fetal cerebral cortex from embryonic‐day 17 C57BL/6JNarl mice was aseptically isolated and dissociated as previously described.^[^ ^41^ ^]^ Neurosphere cultures were prepared in neurobasal medium supplemented with 2% B‐27 supplement, 0.5 mM l‐glutamine, and 1% penicillin‐streptomycin. For the maintenance and expansion of the cultures, neurobasal medium was supplemented with 20 ng mL^−1^ each of basic fibroblast growth factor (bFGF) and epidermal growth factor (both from Gibco, USA), in a humidified incubator with 5% CO_2_ at 37 °C. To create clonal and low‐density cultures, primary neurospheres grown at a high density were dissociated into a single‐cell suspension and seeded into 6‐well plates, at a cell density of 1×10^5^ cells per mL of medium. Newly formed neurospheres were counted after 7 d. Clonal cultures were passaged every 7 d. The self‐renewal potential was quantified as the number of secondary neurospheres generated per primary neurosphere. In addition, the capacity for secondary neurosphere formation was measured by dissociating primary neurospheres back into single cells.^[^ ^42^ ^]^ Clonally derived neurospheres were counted (images of three fields were obtained per well) and their diameters were measured using bright‐field and image analysis software. Cell counting and population doubling were performed as previously described.^[^ ^43^ ^]^

### RNA‐Sequencing

Neurospheres were treated with EXO and EXO‐PD‐L1‐HGF for 24 h, and total RNA extraction was performed using PureLink RNA Mini Kit (Thermo Fisher Scientific, USA), according to the manufacturer's instructions. Total RNA lysates from the neurospheres subjected to EXO and EXO‐PD‐L1‐HGF treatments were used to prepare each RNA‐sequencing library using an HiSeq 2000 sequencer (Illumina) at the Genomic Core facility of the Mayo Clinic. Paired‐end sequencing libraries were prepared using a TruSeq Stranded Total Sample Preparation Kit (Illumina) at the Mayo Clinic Sequencing Core Facilities, followed by quality control, cluster generation, and sequencing on a HiSeq 2000 platform. The results of the RNA‐sequencing were delivered as binary alignment and map (BAM) files, which were converted to FASTQ format using Picard. FASTQ files were aligned to the hg19 human reference genome using Top Hat 2.0.14. The expression values were calculated using featureCounts v1.4.6, and differential expression analysis was performed using DESeq2.

### Depletion of Phospho‐STAT3 Expression Using STAT3 Inhibitor

PCCs were seeded at a density of 3 × 10^5^ cells per well into a 24‐well plate overnight and pretreated with 20 µM Stat3 inhibitor (Cat# S3I‐201, Santa Cruz Biotechnology, USA) for 6 h, in a humidified incubator with 5% CO_2_ at 37 °C. Following treatment, the cells were divided into three treatment groups, including control, EXO (20 µg), and EXO‐PD‐L1‐HGF (20 µg), and subjected to the respective treatments for 24 h, at 37 °C in a humidified incubator with 5% CO_2_, to determine the phosho‐STAT3 expression by means of western blot.

### Preparation of Lentiviruses for shFOXO3

Mouse FOXO3 short hairpin RNA (shFOXO3) was purchased from the RNAi Core Center (Academia Sinica, Taiwan). FOXO3 shRNA or scrambled shRNA was cloned into the pLKO1 expression vector. Subsequently, these constructs were co‐transfected with lentiviral packaging plasmids (pCMV‐ΔR8.91 and pMD.G) into HEK‐293T cells using Lipofectamine 3000 transfection reagent (Cat# L3000001, Thermo Fisher Scientific, USA). After 72 h of transduction, the lentiviral particles containing shFOXO3 (LV‐sh‐FOXO3) were collected using 0.45‐µm filters and concentrated by means of centrifugation at 4000 × *g* for 15 min, followed by a second centrifugation at 1000 × *g* for 2 min at room temperature. The concentrated virus was then stored at −80 °C for further experiments.

### Immunocytochemistry

PCCs and NPCs were washed with sterile 1× PBS and fixed in 4% paraformaldehyde for 15 min at room temperature. After washing with PBS, the fixed cells were permeabilized using 0.3% Triton X‐100 for 30 min. Subsequently, the cells were blocked with a solution containing 300 µM glycine and 2% FBS in PBS. Following blocking, the cells were incubated overnight at 4 °C with primary antibodies against GFAP (clone: G‐A‐5, 1:400, Sigma, USA), microtubule‐associated protein 2 (MAP2, clone: AP20, 1:300, Merck Millipore, USA), nestin (clone: 10C2, 1:500, Merck Millipore, USA), and SOX2 (Clone: 245610, 1:500, R&D Systems, USA). After three washes with PBS, the cells were incubated with FITC‐ or phycoerythrin‐conjugated secondary antibodies for 60 min at room temperature. The cellular nuclei were stained with DAPI. Immunohistochemical images were analyzed using a SP2 Confocal Spectral Microscope.

### Animal Brain Ischemia/Reperfusion Model and Treatment Protocol

Adult male C57BL/6 mice (25–30 g) were subjected to two‐vessel ligation. All surgical procedures were performed using sterile/aseptic techniques, in compliance with the Institutional Guidelines for Animal Research and were approved by the Institutional Ethical Committee for Animal Research. The mice were anesthetized with 2% isoflurane in oxygen. Ligation of the right middle cerebral artery (MCA) and right common carotid arteries was performed as previously described.^[^ ^44^ ^]^ The right common carotid arteries were clamped using nontraumatic arterial clips. Using a surgical microscope, a 2 × 2 mm^2^ craniotomy was performed at the junction of the zygoma and squamosal bone. The right MCA was ligated using a l0–0 nylon suture. Cortical blood flow was continuously monitored using a laser Doppler flowmeter (PF‐5010, Periflux System) in the anesthetized animals. A 1‐mm burr hole was made in the right frontoparietal region to place the photodetectors. A probe (0.45 mm diameter) was stereotaxically inserted into the cortex (l.3 mm posterior, 2.8 mm lateral to the bregma, and l.0 mm below the dura). After 120 min of ischemia, the MCA sutures and arterial clips on the common carotid arteries were removed to initiate reperfusion. Experimental mice were then injected with EXO or EXO‐PD‐L1‐HGF (200 µg in 100 µL saline) or control (100 µL saline) into the right femoral vein, at 30 min after reperfusion, through a 26‐gauge syringe. The core body temperature was monitored using a thermistor probe during anesthesia and maintained at 37 °C using a heating pad. After recovery, the body temperature was maintained at 37 °C with a heat lamp.^[^ ^45^ ^]^

### Stereotaxic Injection of LV‐sh‐FOXO3

Experimental mice were intracerebrally injected with LV‐sh‐FOXO3 (50 µL) at 30 min after MCA ligation, using a 30‐gauge Hamilton syringe (Hamilton Company, USA). The injections were targeted at two cortical areas adjacent to the right MCA, positioned 2.0–3.0 mm below the dura. The approximate coordinates for these injection sites were as follows: 0.5–1.0 mm anterior to the bregma and 1.5–2.0 mm lateral to the midline, and 1–l.5 mm posterior to the bregma and 2–2.5 mm lateral to the midline. After each injection, the needle was left in place for five min and a piece of bone wax was applied to the skull defects, to prevent leakage of the injected solution.

### Nestin‐GFP‐Transgenic Mice

Nestin‐GFP transgenic mice were kindly provided by Dr. Kempermann and Dr. Yamaguchi.^[^ ^46^ ^]^ The mice were maintained as homozygotes for the transgene in a C57BL/6 genetic background. All mouse colonies were bred and genotyped in a specific pathogen‐free animal research facility at the China Medical University, under a 12 h/12 h light/dark cycle. All animal breeding and experimental procedures were approved by the Animal Care and Use Committee of China Medical University. The genotyping result of Nestin‐GFP mice in Figure S12a in the Supporting Information.

### FOXO3 Knockout Mice

Heterozygous FOXO3 (*FOXO3^+/–^*) mice (strain B6;129P2‐Foxo3<tm1Nmt>) were obtained from RIKEN (strain number: RBRC03482, Japan). Generation and genotyping of the mice were performed as previously described.^[^ ^47^ ^]^ Normal littermate (FOXO3‐NL) and knockout (*FOXO3^–/–^*) mice were housed in a specific pathogen‐free environment at China Medical University, under a 12 h/12 h light/dark cycle. All the experimental protocols were approved by the University Committee on Animal Research at the University of Rochester. The genotyping result of FOXO3‐knockout mice in Figure S12b in the Supporting Information.

### Neurological Behavioral Measurements

Behavioral assessments were performed 5 d before cerebral ischemia and at 0, 7, 14, 21, and 28 d after exosome treatment. The tests included rotarod treadmill and beam walking tests. The baseline test scores were recorded to normalize the values obtained after cerebral ischemia. An automated rotarod treadmill consisting of a roller with a 5 cm diameter suitably machined to provide grip and a power source to turn the roller was used. Five circular separators divided the rod into equal‐sized compartments (5 cm in length each), allowing four rats to be on the treadmill simultaneously. The time spent by each mouse (control, EXO, and EXO‐PD‐L1‐HGF groups) on the rotarod (20–25 rpm) was measured using timers. These timers detected the time at which the rat fell off the treadmill onto the plate below, or if it remained on the rod for up to two min. The balance beam was a rod 160 cm in length and 2.5 cm in diameter. A plastic platform (7×4 cm^2^) was set at one end of the rod as the starting point, while a black plastic box (15×15×8 cm^3^) was placed at the other end of the rod, as a motivating nest for the animal to cross the beam. The apparatus was suspended 90 cm above a cushion (to protect the fallen animals from injury) and positioned 50 cm away from the wall. The time required to traverse the beam was recorded.

### TTC Staining

Three days after reperfusion, the animals were intracardially perfused with saline. The brain tissue was then removed, immersed in cold saline for five min, and sliced into 2‐mm thick sections (four slices per mouse). These brain slices were subsequently incubated in a 20 g L^−1^ solution of TTC (Research Organics, USA) dissolved in saline, at 37 °C for 30 min. After incubation, the slices were transferred to a 4% formaldehyde solution for fixation. The area of infarction in each slice was measured using a digital scanner. The infarction volume was calculated by multiplying the average slice thickness (2 mm) by the sum of the infarcted areas in all the brain slices examined. To account for brain edema, the total infarcted area was expressed as a percentage of the contralateral half of the brain.^[^ ^48^ ^]^ This percentage was calculated by dividing the sum of the infarct areas by the sum of the contralateral hemibrain areas.

### In Vivo Biodistribution Analysis

The lipophilic dye DiD (Thermo Fisher Scientific, USA) was used to label the membrane of exosome. For DiD labeling, 200 µg of exosomes were incubated with DiD for 1 h at room temperature. After staining, the free dye was wash‐out by Total Exosome Isolation reagent. Following the induction of stroke, the mice were randomly grouped and injected with DiD‐stained EXO or EXO‐PD‐L1‐HGF via the femoral vein. At various time intervals (4, 24, and 72 h), the mice were anesthetized with 2% isoflurane in oxygen and fluorescence images were acquired using an in vivo imaging system with Living Image 3.0 software (Xenogen) (excitation: 745 nm and emission: 830 nm). After 72 h, the mice were euthanized and the ROI tool was used to quantify the fluorescence signal.

### Bromodeoxyuridine (BrdU) Labeling and Immunohistochemistry

BrdU, a thymidine analog incorporated into the DNA of dividing cells during S phase, was used for mitotic labeling (Sigma, USA). The labeling protocol has been described previously.^[^ ^49^ ^]^ Briefly, a pulse‐labeling method was used to observe the time‐course of the proliferating cells in the brain after cerebral ischemia. The experimental animals were intraperitoneally injected with BrdU (50 mg kg^−1^) every 4 h for 12 h before sacrifice. A cumulative labeling method was used to examine the population of proliferative cells during the 14 d of cerebral ischemia. Animals received daily injections of BrdU (50 mg kg^−1^, intraperitoneal) for 14 consecutive days, starting on the day after MCA ligation. The animals were sacrificed 14 d after the last injection. BrdU immunostaining with a specific antibody against BrdU (1:400, Boehringer Mannheim) and quantification of BrdU‐immunoreactive cells have been described previously.^[^ ^49^ ^]^ For BrdU immunostaining, DNA was denatured by incubating each section in 50% formamide in 2× standard saline citrate, at 65 °C for 2 h, followed by incubation in 2 N HCl, at 37 °C for 30 min, and finally rinsing in 0.1 m boric acid at pH 8.5. The immunostaining procedure was performed by staining with the appropriate diluted antibodies to BrdU (1:400, Thermo Fisher Scientific, USA) at room temperature for 1 h, followed by treatment with specific secondary antibodies conjugated with Cy3 (1:500, goat antirabbit IgG) or FITC (1:500, goat antimouse IgG) (both from Jackson ImmunoResearch), and double immunostaining to demonstrate their co‐localization in one cell, using the 510 confocal laser‐scanning microscope (Zeiss, Germany).

### Immunohistochemical Assessment

Animals were anesthetized with 2% isoflurane in oxygen and their brains were fixed by means of transcardial perfusion with saline, followed by perfusion and immersion in 4% paraformaldehyde. Tissue samples were harvested, fixed by immersion in 4% paraformaldehyde, dehydrated in 30% sucrose, and frozen on dry ice. Coronal sections (6‐µm thick) were cut by means of a cryostat, stained with hematoxylin‐eosin, and observed by means of light microscopy (E600, Nikon, Japan). Each coronal section was first stained with an antibody against neural/glial antigen 2 (NG2; 1:200), GFAP (1:500), DCX (1:300), stromal cell‐derived factor (SDF)−1α (1:200), and GFP (1:200) (all from Merck Millipore, USA), followed by treatment with specific secondary antibodies conjugated with Cy3 (1:500, goat antirabbit IgG) or FITC (1:500; goat antimouse IgG). Double immunostaining was performed to demonstrate the co‐localization of Cy3 and FITC in a single cell using confocal laser‐scanning microscopy. For semi‐quantification of fluorescent cells in the peri‐infarct region, measurements and colocalization were analyzed by means of a semi‐random setting in the four square regions over the infarct core, using the Zeiss 510 confocal laser‐scanning microscope, as described previously.

### Flow Cytometry for T, Dendritic, Macrophage, and Natural Killer Cell Populations In Vivo

Cells from the peri‐infarct brain tissue and splenocyte suspension were washed with sterile 1× PBS containing 2% bovine serum albumin and 0.1% sodium azide. Subsequently, the cells were incubated with the respective antibodies and reagents (PD‐L1, CD3, CD8, CD4, CD11c, CD19, CD25, CD45, CD80, CD86, Foxp3, CD44, CD45, CD11b, F4/80, interferon‐γ (IFN‐γ), 7‐AAD, tumor necrosis factor‐α (TNF‐α), IL‐10, NK1.1, CD122, and CD206) from BD conjugated with appropriate fluorescent dyes before analysis. Cells were stained with mouse IgG1 isotype control antibodies. The gating strategy was based on the correct justification of the first gate, exclusion of doublets by forward scatter (FSC)‐A and FSC‐H, exclusion of dead cells, and further selection as 7‐AAD^+^/CD45^+^ according to previous literature,^[^ ^50^ ^]^ using an Attune NxT Flow cytometer with NxT Software and FlowJo Software v.7.6. The results are expressed as the percentage of positively stained cells relative to the total cell number.

### Statistical Analysis

All measurements were performed in a blinded manner. Results are presented as mean ± standard deviation of three independent experiments. Statistical analysis was performed using the Mann–Whitney *U*‐test for comparisons between two groups and one‐way analysis of variance followed by Tukey's post hoc test for comparisons between more than two groups. Statistical significance was considered significant at *p *< 0.05.

## Conflict of Interest

The authors declare no conflict of interest.

## Supporting information

Supporting Information

## Data Availability

The data that support the findings of this study are available from the corresponding author upon reasonable request.
