# Supplementary material for: Role of STAT3‐FOXO3 Signaling in the Modulation of Neuroplasticity by PD‐L1‐HGF‐Decorated Mesenchymal Stem Cell‐Derived Exosomes in a Murine Stroke Model
Source: Adv Sci (Weinh). 2024 Jul 25;11(36):2404882. doi: 10.1002/advs.202404882 (PMC11423231; doi:10.1002/advs.202404882)
Supplement: Supplementary file 1 — Supporting Information [file ADVS-11-2404882-s001.docx]

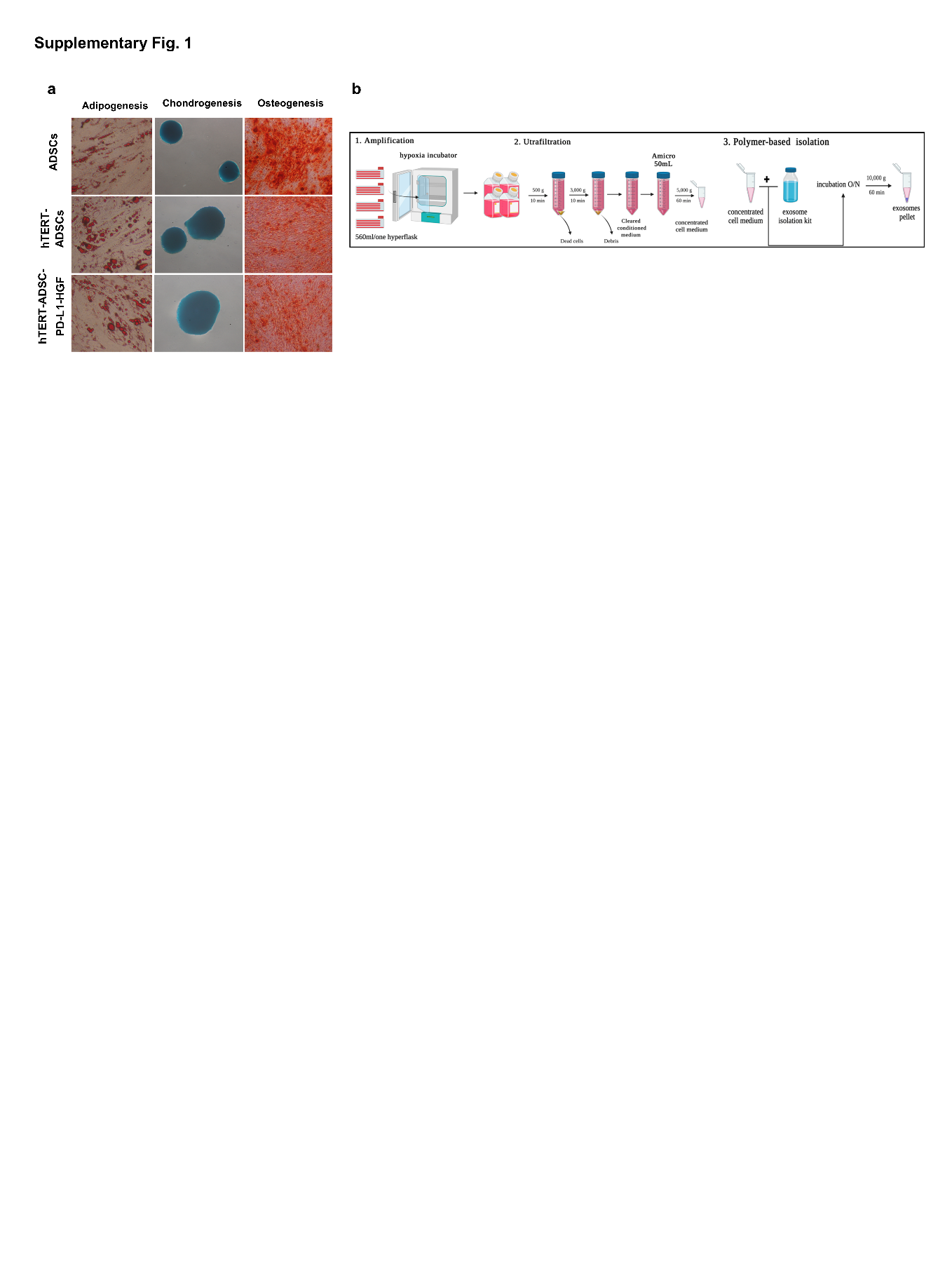
**Figure S1**

**Figure S1.** (a) Representative histological images of the trilineage differentiation of adipocyte, chondrocyte, and osteocyte in ADSCs, hTERT-ADSCs, and hTERT-ADSC-PD-L1-HGF cells. (b) Optimization of the protocol of production and isolation of exosomes.

**Figure S2**

**
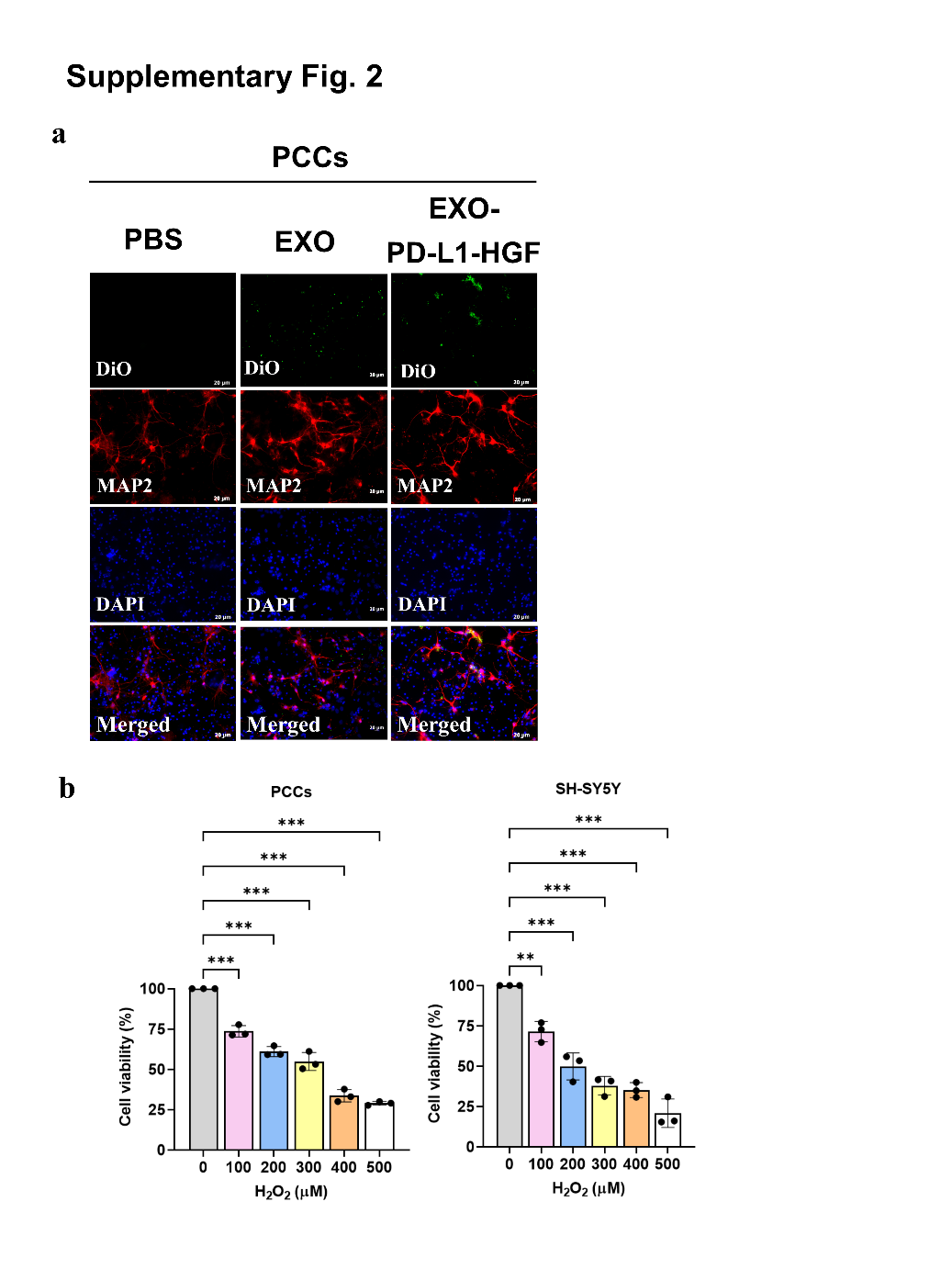

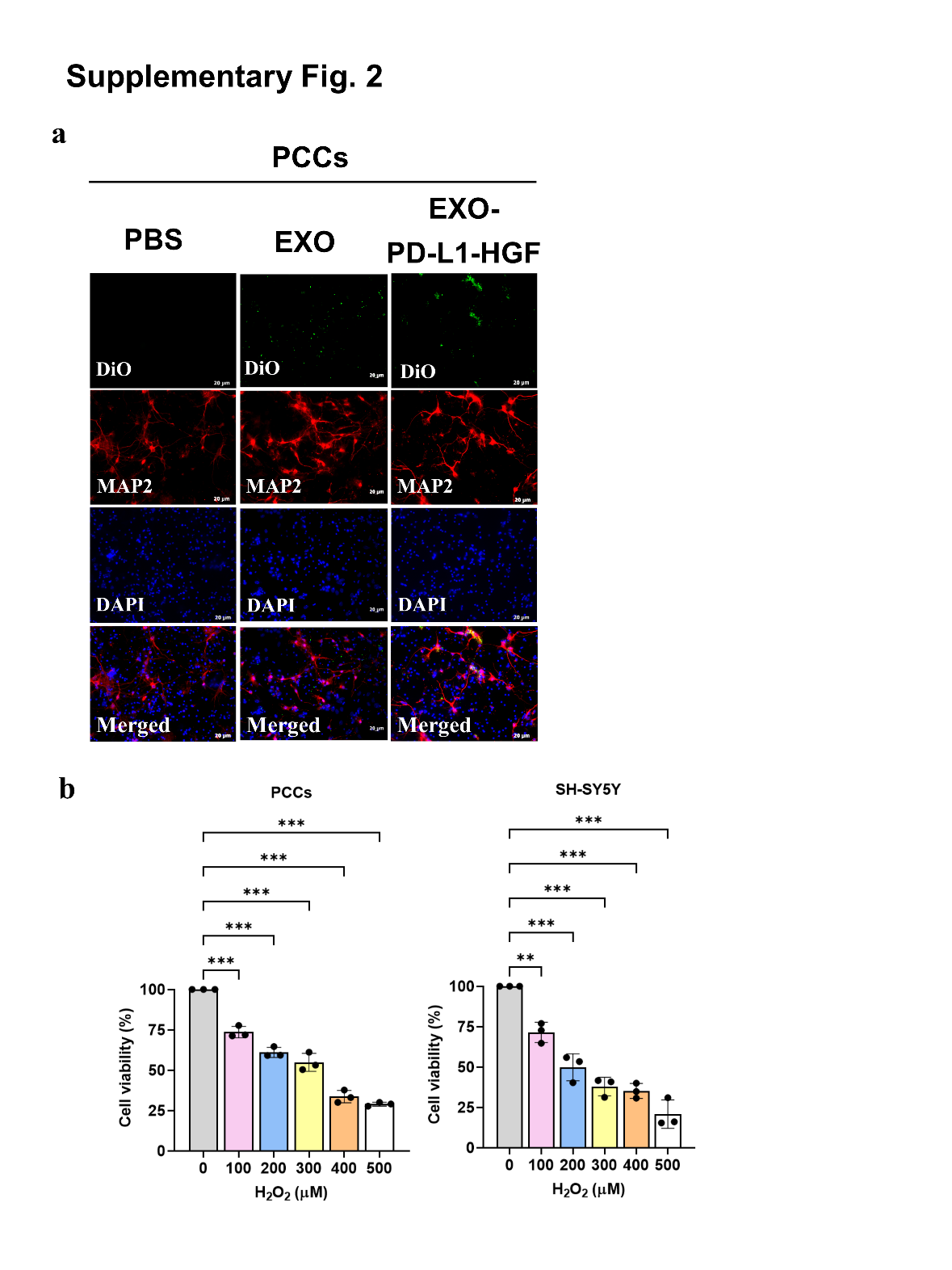
**

**Figure S2.** (a) Representative immunocytochemical images of endocytosis of DiO-labeled exosomes in MAP2-expressed PCCs. (b) Quantitative analysis of H_2_O_2_ concentration-dependently induced cell death in PCCs and SH-SY5Y cells using CCK-8 analysis.

**Figure S3**

**
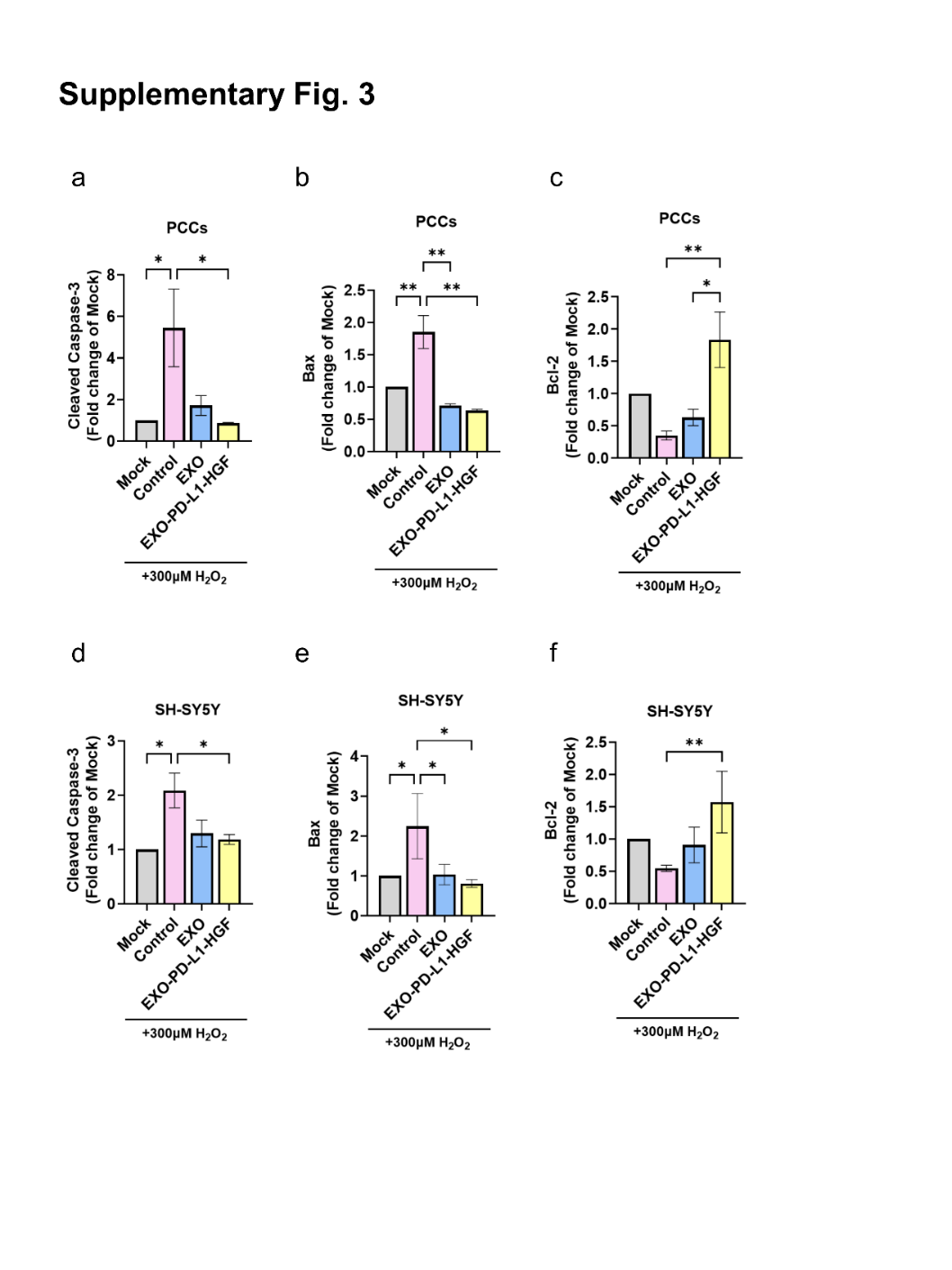
**

**Figure S3.** Quantitative results of (a, d) cleaved caspase-3, (b, e) Bax, and (c, f) Bcl-2 protein expression under EXO-PD-L1-HGF treatment in H_2_O_2_-induced neuronal apoptosis in PCCs and SH-SY5Y cells using western blot analysis.

**Figure S4**

**
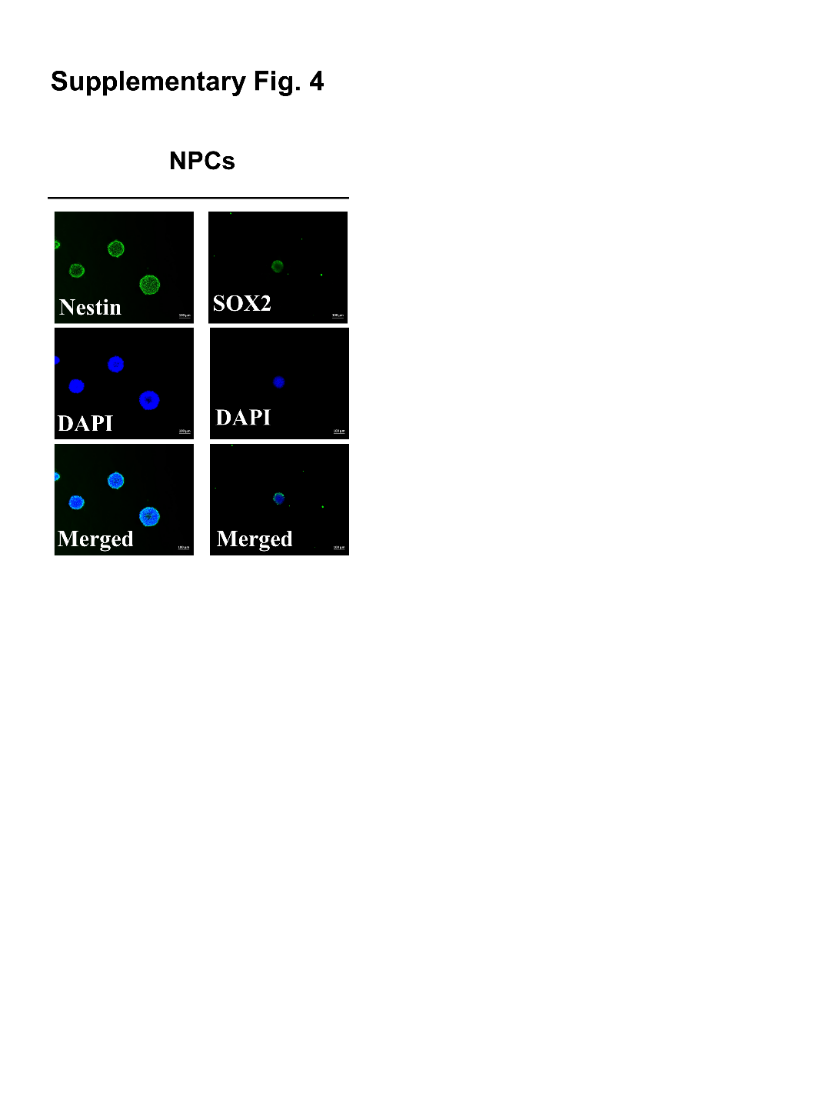
**

**Figure S4.** Representative immunofluorescent images of the neurospheres markers including nestin and SOX2.

**Figure S5**

**
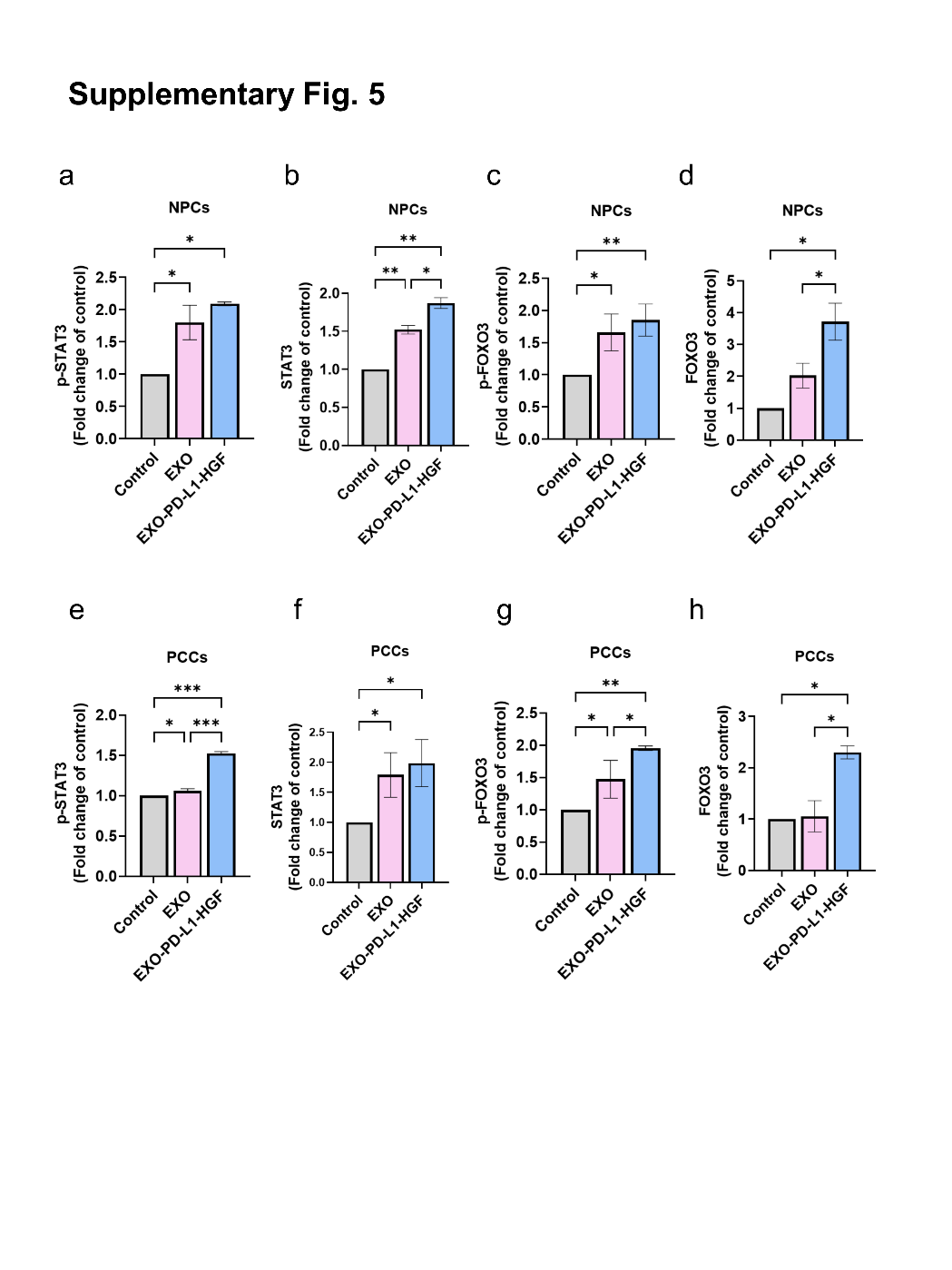
**

**Figure S5.** Quantitative results of (a, e) phospho-STAT3, (b, f) STAT3, (c, g) phospho-FOXO3, and (d, h) FOXO3 protein expression though EXO and EXO-PD-L1-HGF treatment in NPCs and PCCs using western blot analysis.

**Figure S6**

**
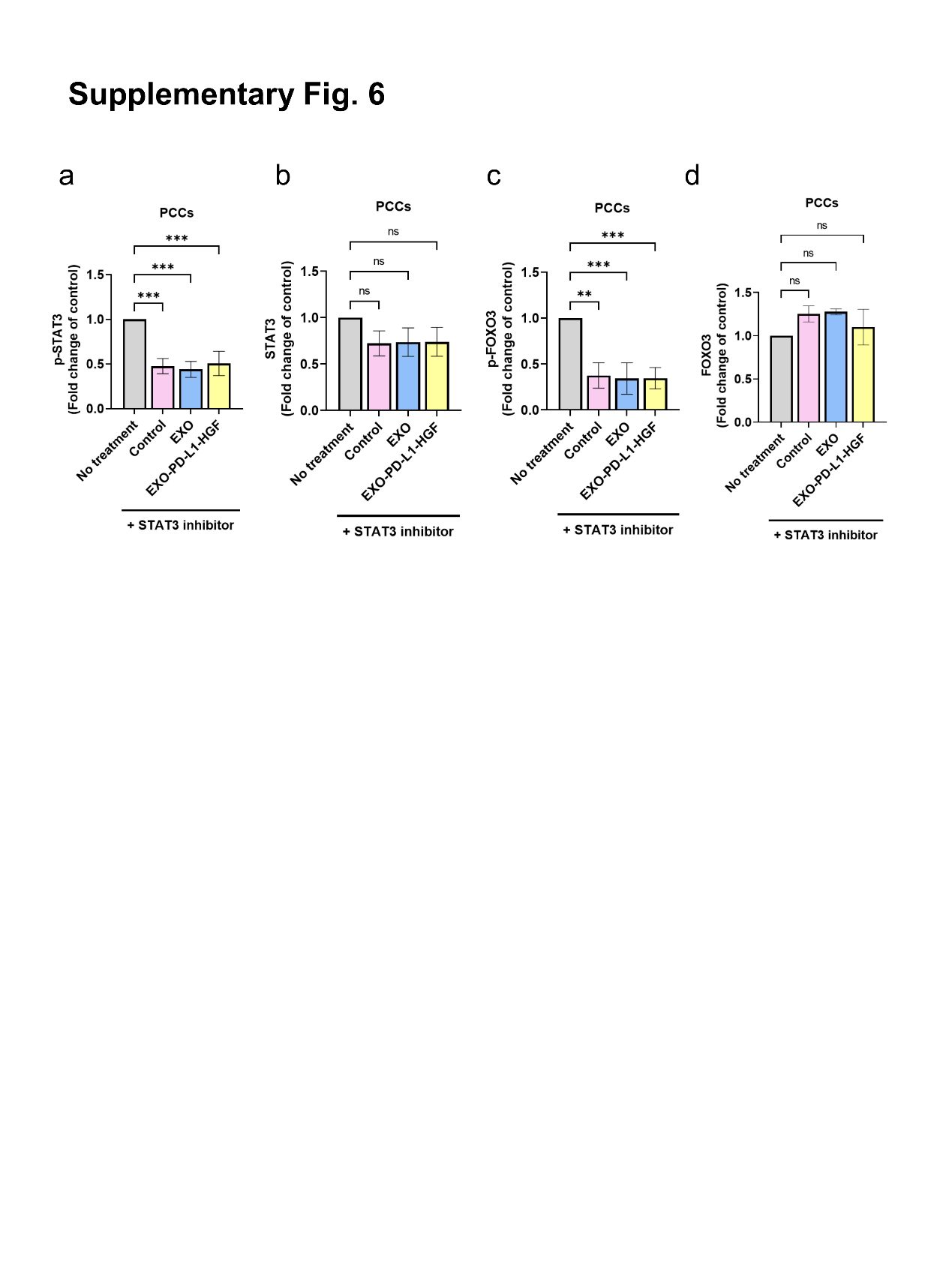
**

**Figure S6.** Quantitative results of (a) phospho-STAT3, (b) STAT3, (c) phospho-FOXO3, and (d) FOXO3 protein expression under EXO and EXO-PD-L1-HGF treatment with STAT3 inhibitor using western blot analysis.

**Figure S7**

**
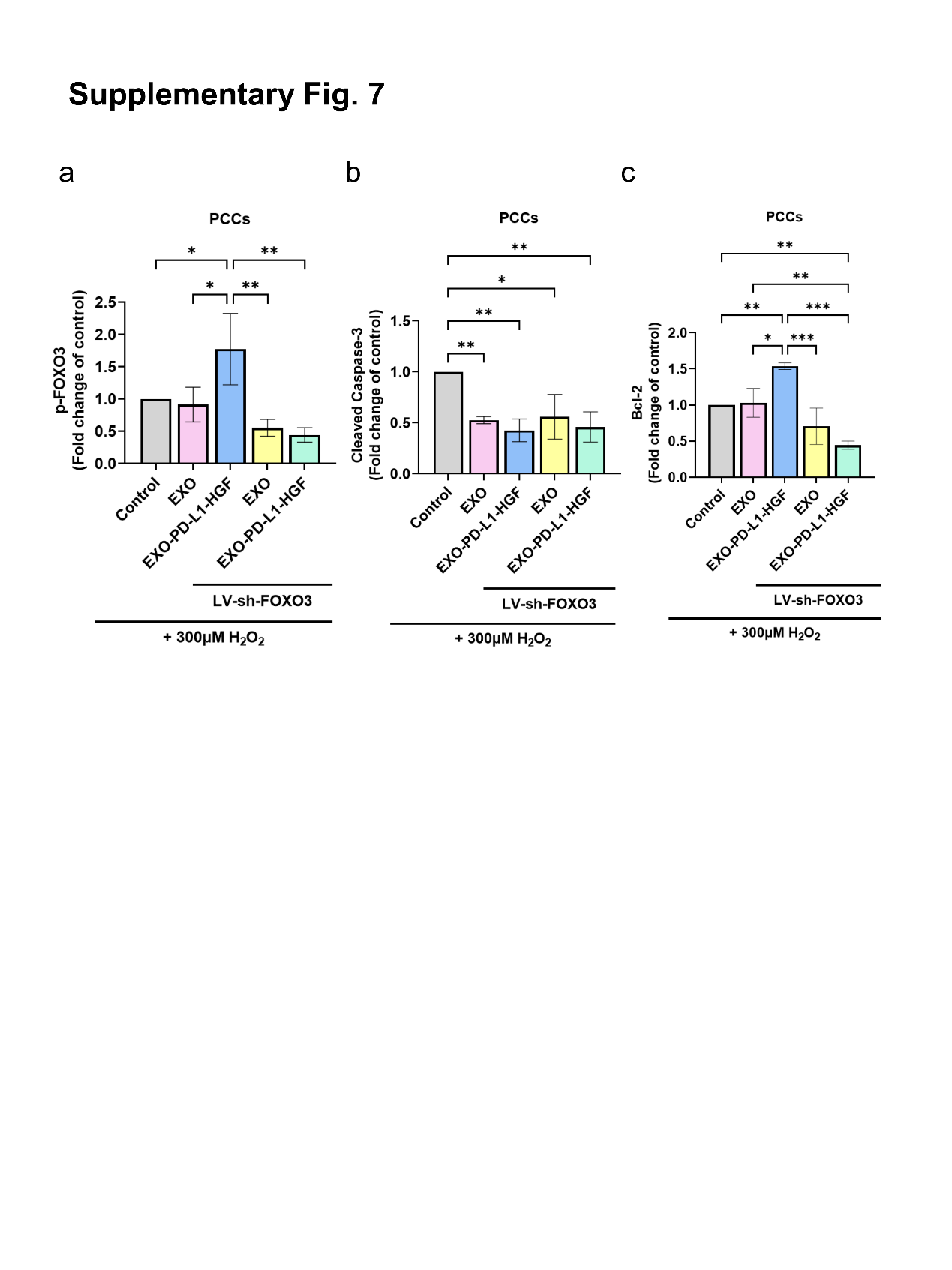
**

**Figure S7.** Quantitative results of (a) the phospho-FOXO3, (b) cleaved caspase-3, and (c) Bcl-2 protein expression under EXO-PD-L1-HGF with FOXO3 knockdown by lenti-viral infection (LV-sh-FOXO3) in H_2_O_2_-induced neuronal apoptosis using western blot analysis.

**Figure S8**

**
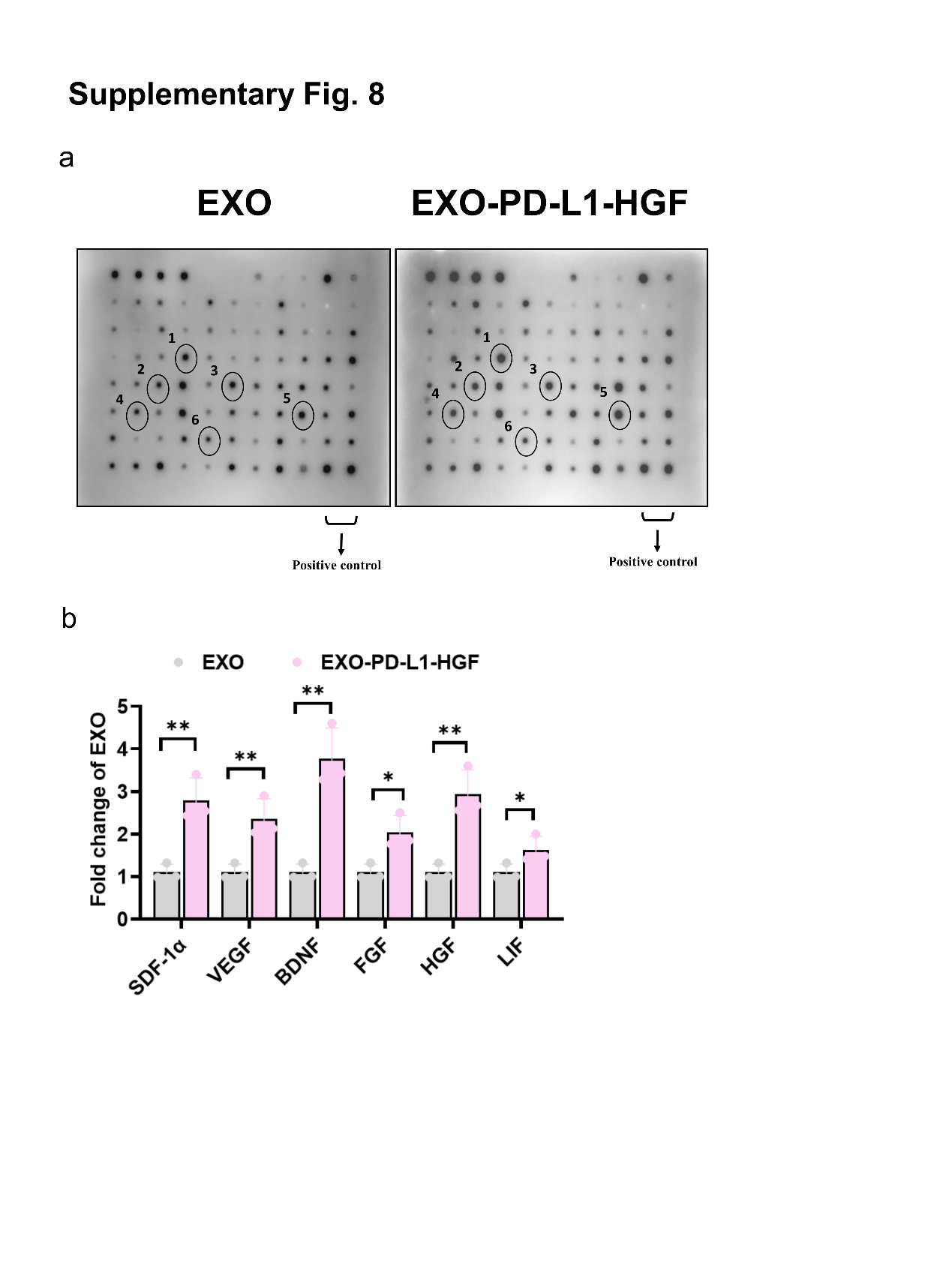
**

**Figure S8.** (a) Representative dot images of cytokine array (total 80 items) after EXO and EXO-PD-L1-HGF administration for picking up of 6 specific cytokines (#1: SDF-1α, #2: VEGF, #3: BDNF, #4: FGF, #5: HGF, #6: LIF) marked by black circle. (b) Quantitative results for comparison with the levels of SDF-1α, LIF, BDNF, VEGF, FGF, and HGF after the treatment of EXO and EXO-PD-L1-HGF using cytokine array analysis.

**Figure S9**

**
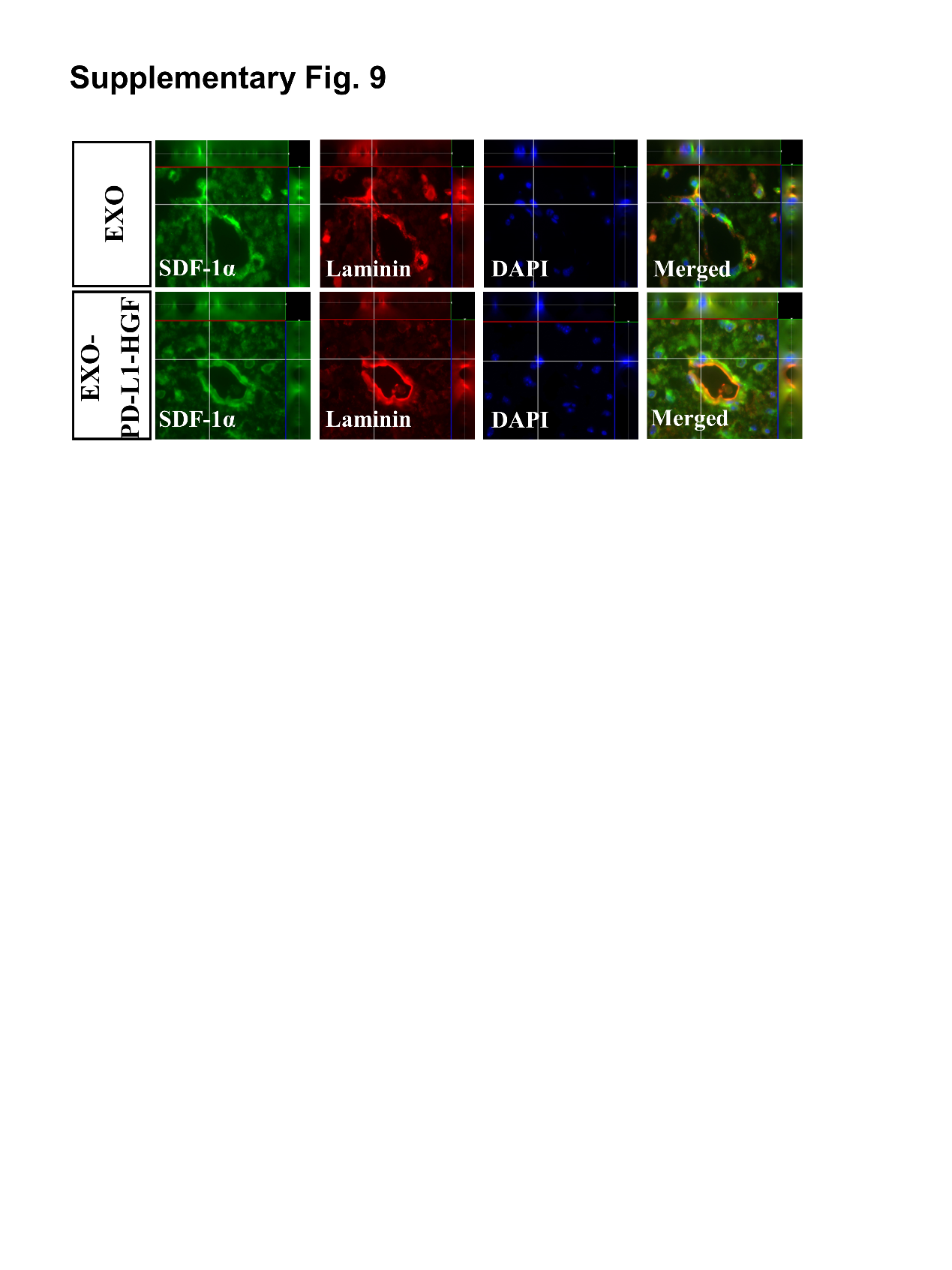
**

**Figure S9.** Representative immunohistochemical image of the SDF-1α expression in co-localization with the laminin^+^ vessels in EXO and EXO-PD-L1-HGF treatment after ischemic stroke

**Figure S10**

**
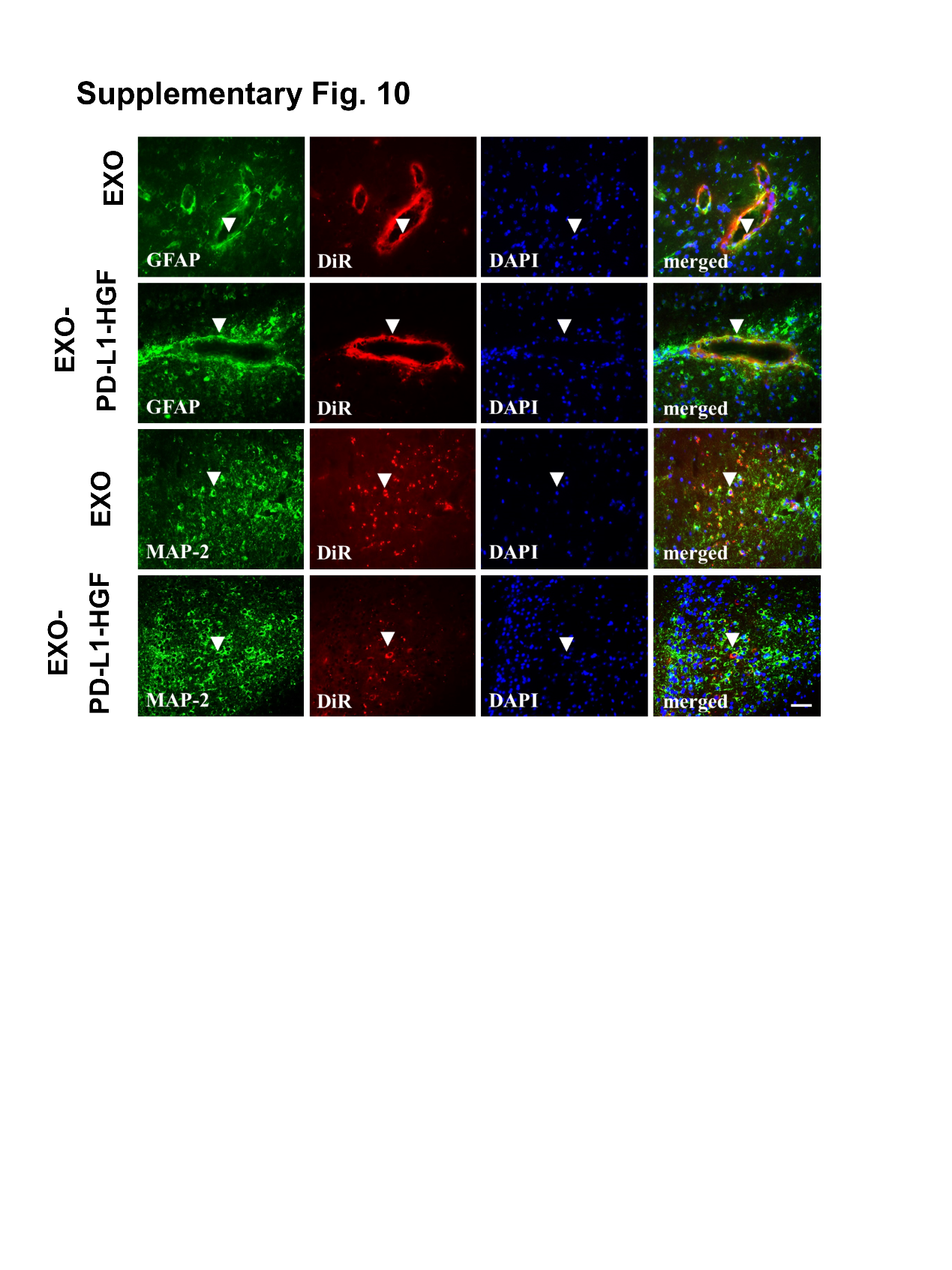
**

**Figure S10.** Representative immunohistochemical images of DiR-labeled EXO and EXO-PD-L1-HGF in co-localized with GFAP^+^ and MAP-2^+^ neuroglial cells in ischemic brain.

**Figure S11**

**
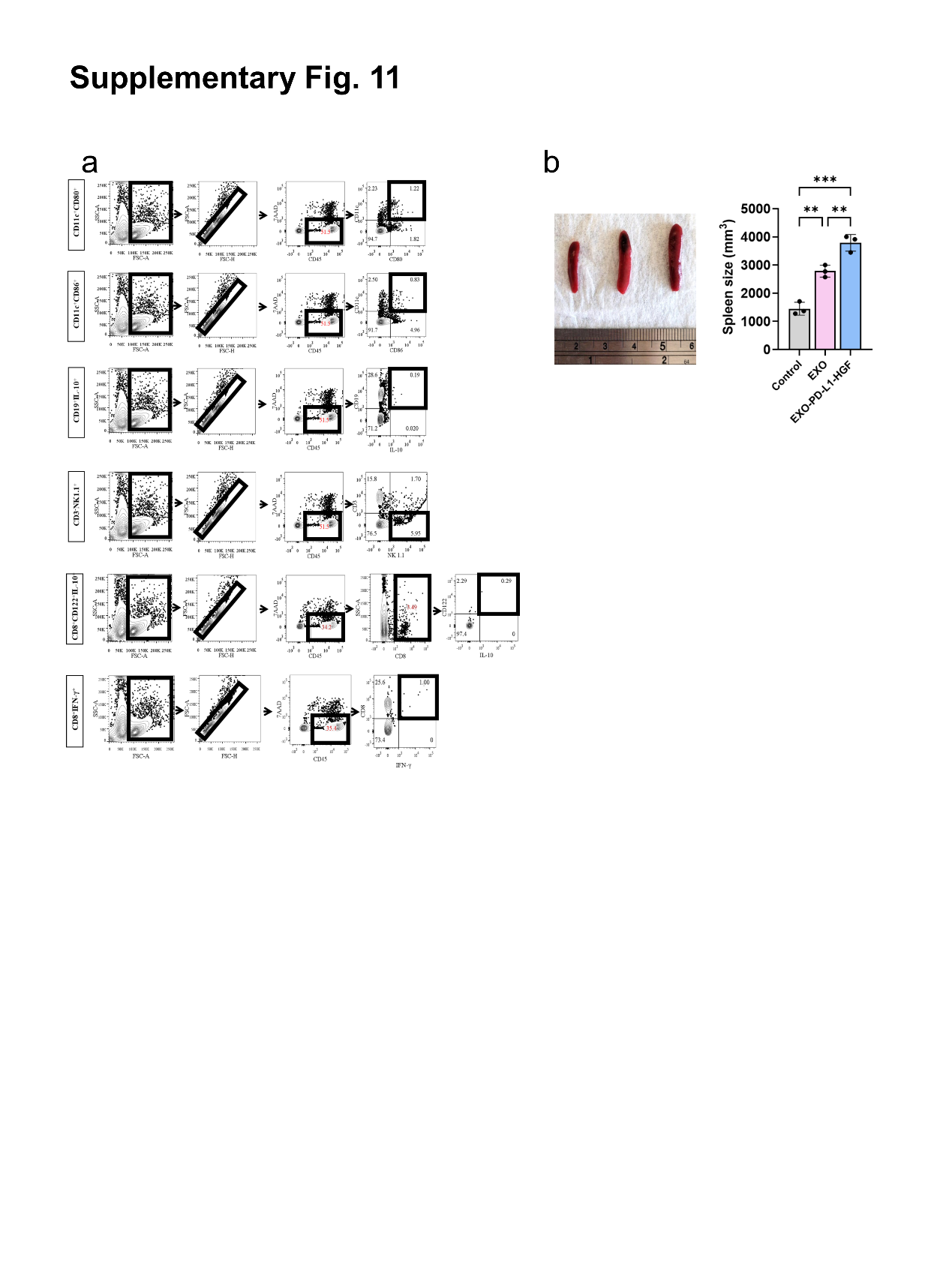
**

**Figure S11.** (a) Representative gating strategy for flowcytometric analysis of the activated dendritic cells (CD11c^+^CD80^+^, CD11c^+^CD86^+^), cytotoxic T cells (CD8^+^IFN-γ^+^), natural killer cells(CD3^–^NK1.1^+^), regulatory B cells (CD19^+^IL-10^+^), and Treg cells (CD8^+^CD122^+^IL-10^+^). (b) Representative gross images of spleen morphology after control, EXO and EXO-PD-L1-HGF treatment (left panel). Quantitative analysis of spleen size after EXO and EXO-PD-L1-HGF treatment in stroke mice (right panel).

**Figure S12**

**
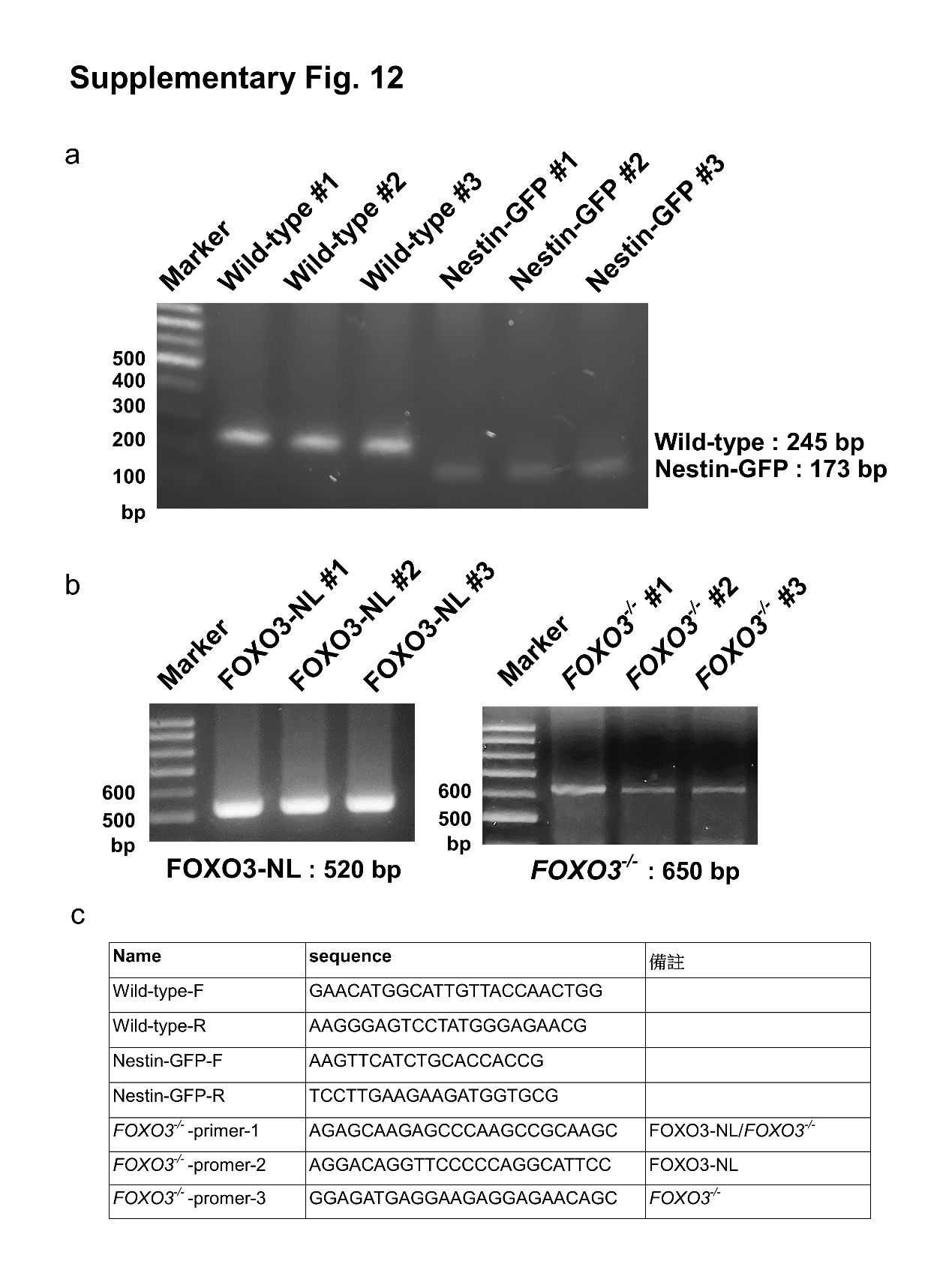
**

**Figure S12.** (a) Representative genotyping result of nestin-GFP mice using specific primers for PCR analysis. The PCR product of nestin-GFP mice was 173 bp, and the wild-type was 245bp. (b) Representative genotyping result of FOXO3-NL and FOXO3 knockout mice (*FOXO3^-/-^*) using PCR analysis. The PCR product of *FOXO3^-/-^* mice was 650 bp, and the FOXO3-NL was 520 bp. (c) The table showed the genotyping primer sequences of nestin-GFP mice and *FOXO3^-/-^* mice.

**Table S1**

**
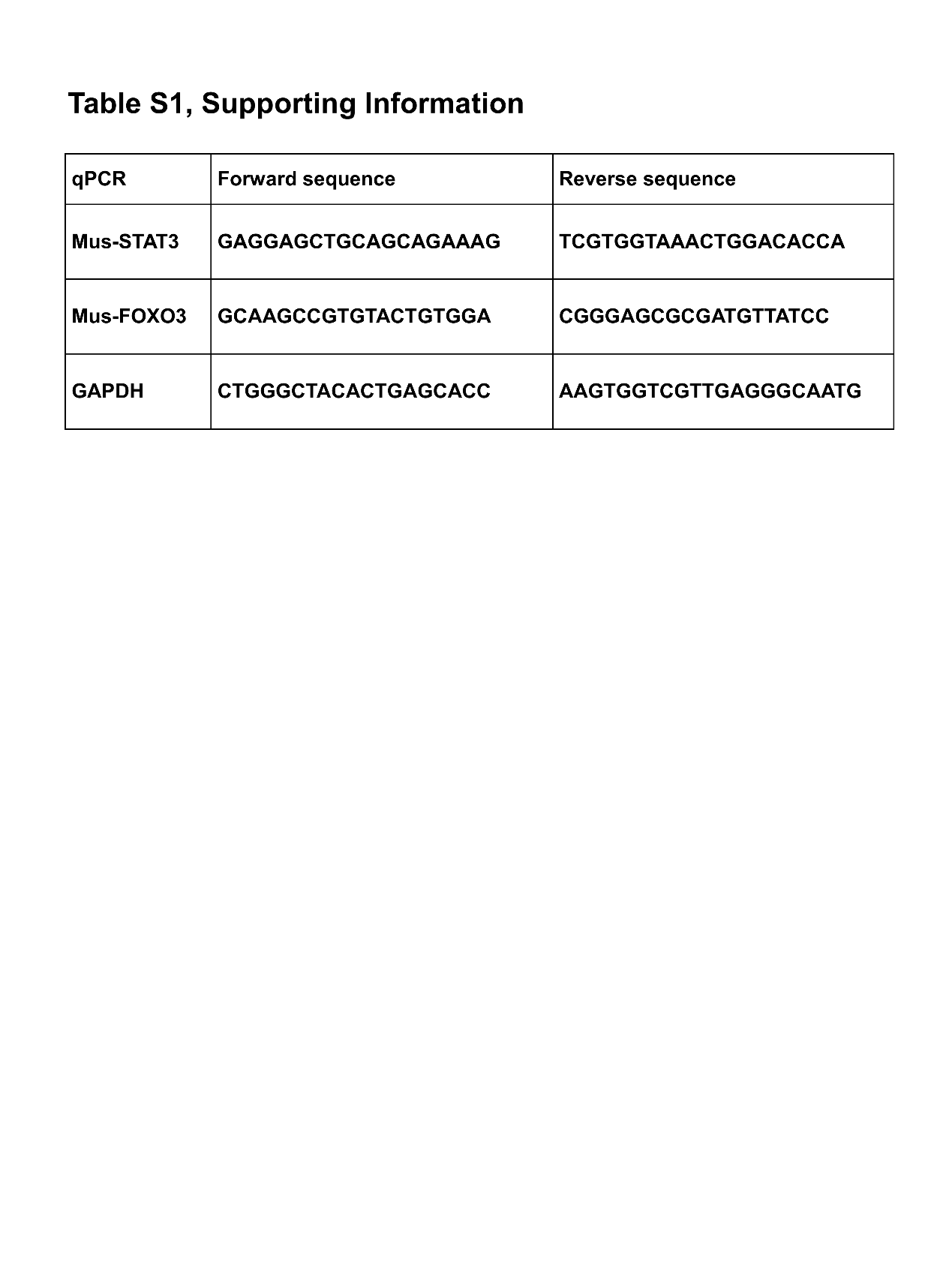
**

**Table S1.** The Real-time Quantitative PCR (qPCR) primer sequences of Mus-STAT3, Mus-FOXO3, and GAPDH.
